# AI‐EXAFS: Physics‐Informed Prediction of EXAFS from Atomic Structure

**DOI:** 10.1002/advs.78226

**Published:** 2026-10-18

**Authors:** Javier Heras‐Domingo, Andrea Ruiz‐Ferrando, Adam H. Clark, Stephan Pollitt, Sharon Mitchell, Javier Pérez‐Ramírez, Núria López

**Affiliations:** ^1^ Institute of Chemical Research of Catalonia (ICIQ‐CERCA) The Barcelona Institute of Science and Technology (BIST) Tarragona Spain; ^2^ Departament de Química Inorgànica i Orgànica (Secció de Química Inorgànica) Institut de Química Teòrica i Computacional (IQTC) Universitat de Barcelona Barcelona Spain; ^3^ Department of Chemistry and Applied Biosciences Institute for Chemical and Bioengineering ETH Zurich Zurich Switzerland; ^4^ NCCR Catalysis Zürich Switzerland; ^5^ Paul Scherrer Institute Villigen Switzerland

**Keywords:** extended X‐ray absorption fine structure, graph neural network, local environment, smart characterization, structure‐to‐spectrum prediction, X‐ray absorption spectroscopy

## Abstract

Understanding atomic structure across chemical systems relies heavily on extended X‐ray absorption fine structure (EXAFS) because of its sensitivity to local atomic environments. However, quantitative interpretation remains inherently iterative, requiring structural parameters or models to be fitted against experimental spectra using explicit multiple‐scattering calculations. Machine‐learning approaches have accelerated EXAFS analysis, yet direct prediction of full spectra from atomistic structure across diverse chemical environments remains challenging. Here, we introduce AI‐EXAFS, a physics‐informed graph neural network that predicts spectra directly from three‐dimensional atomic coordinates. Combining an absorber‐centered graph representation with distance‐modulated message passing and an EXAFS‐specific training objective enables direct learning of structure–spectrum relationships without predefined structural descriptors or manual parameter selection. Trained and evaluated across a dataset of 85,991 transition‐metal complexes with FEFF‐generated spectra, AI‐EXAFS achieves average held‐out test‐set *R*‐factors of 0.043 in k‐space and 0.019 in R‐space, demonstrating accurate structure‐to‐spectrum prediction across a broad range of local chemical environments. AI‐EXAFS establishes a scalable structure‐to‐spectrum framework for EXAFS, enabling direct inference across large sets of candidate structures and providing a foundation for data‐driven prediction across increasingly complex and experimentally relevant environments.

## Graphical abstract

AI‐EXAFS presents a neural network to reproduce EXAFS. Trained on synthetic data and with a careful selection of the settings both for the physical representation of the system and following the experimental heuristic loss functions, the model is capable of reproducing EXAFS spectra for molecules from the tridimensional structure.

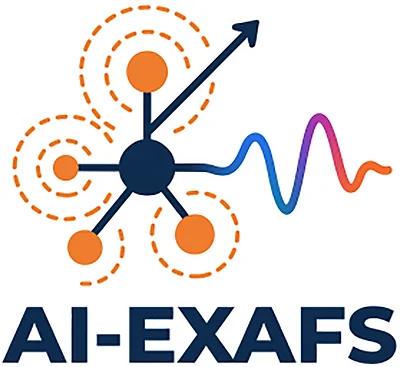

## 1 Introduction

Understanding how local atomic structure impacts chemical behavior is central to materials design, particularly in systems where performance is dictated by isolated sites, short‐range order, defects, disorder, or structural dynamics, rather than long‐range crystallinity [1, 2, 3, 4, 5, 6, 7, 8, 9, 10, 11]. In this context, X‐ray absorption spectroscopy (XAS) has become a key experimental approach because it provides element‐specific information on selected atomic centers across solids, liquids, and molecular systems under a broad range of operating environments [12, 13, 14]. Among XAS techniques, extended X‐ray absorption fine structure (EXAFS) is particularly powerful because it directly probes the local coordination environment surrounding the absorbing atom, including interatomic distances, coordination numbers, local disorder, and structural changes under realistic conditions.

This capability has made EXAFS indispensable across materials and chemical sciences, including heterogeneous catalysts operating under thermal, electrochemical, and photochemical conditions, as well as battery electrodes, disordered solids, and molecular complexes [15, 16, 17]. In catalysis, for example, EXAFS is routinely used to distinguish isolated atoms from clusters and nanoparticles, identify metal–support interactions, follow coordination changes during reaction, and connect local active site structures with catalytic performance [15, 18, 19, 20, 21]. More broadly, EXAFS provides one of the few experimentally accessible routes to quantify local structure in complex materials whose functional behavior cannot be fully understood from diffraction, microscopy, or spectroscopy alone [17].

Despite this broad experimental importance, translating EXAFS spectra into quantitative structural information remains challenging. The measured signal arises from interference between outgoing and backscattered photoelectron waves, involving multiple photoelectron scattering pathways [12, 13, 14]. Consequently, the EXAFS response depends not only on absorber–scatterer distances, but also on the geometric arrangement and connectivity of neighboring atoms, which influence multiple‐scattering contributions. Quantitative interpretation therefore relies on iterative workflows in which candidate atomic structures are repeatedly compared with theoretical spectra, most commonly generated using multiple‐scattering simulations such as FEFF [20, 22, 23]. Although these approaches provide high‐fidelity scattering calculations, they remain expert‐intensive and difficult to scale because structural interpretation requires iterative spectral evaluation, parameter refinement, and validation of physically meaningful solutions. These demands become particularly limiting for time‐resolved, operando, and high‐throughput EXAFS measurements, where large spectral series and multiple plausible structural motifs must be analyzed consistently across many related datasets [15, 16, 24, 25, 26, 27].

Against this background, artificial intelligence (AI) has emerged as a powerful framework for XAS analysis, particularly in the XANES regime, where data‐driven approaches have enabled spectral decomposition, species identification, and extraction of chemical and structural descriptors from modern datasets [28, 29, 30, 31, 32, 33, 34, 35]. Progress in the EXAFS regime, however, has remained more limited. Its highly oscillatory signal distributes structural information across many interfering scattering contributions, complicating representation through simple descriptors. Consequently, many existing machine‐learning approaches learn physically meaningful but radially averaged structural descriptors from EXAFS spectra, such as radial or partial radial distribution functions [18, 36, 37, 38, 39, 40], coordination numbers, or shell‐averaged bonding environments [41, 42, 43, 44, 45, 46, 47], whereas direct learning from explicit three‐dimensional atomic representations remains less explored. While effective within constrained chemical or structural families, many existing approaches are trained and evaluated on comparatively narrow domains, such as specific metals, alloy compositions, nanoparticle classes, or predefined structural motifs. This leaves direct learning of the full EXAFS response from diverse three‐dimensional atomic structures largely unexplored. More general graph‐based models provide a natural way to represent atomic environments [48, 49], but conventional molecular and materials graphs are typically based on chemical connectivity or interatomic proximity and are not specifically designed around the absorber‐centered nature of the EXAFS signal. Adapting graph‐based learning to this problem therefore requires a representation and architecture tailored to the absorbing center and to the structural relationships that shape its scattering response [49].

Here, we introduce AI‐EXAFS, a physics‐informed graph neural network (GNN) that predicts EXAFS spectra directly from three‐dimensional atomic structures. The model combines an absorber‐centered graph representation, distance‐modulated message passing, and EXAFS‐specific optimization objectives to learn direct structure–spectrum relationships without predefined structural descriptors or manual parameter selection.

AI‐EXAFS is trained on transition‐metal complexes, whose well‐defined coordination environments provide broad chemical and structural diversity around the absorbing center [50, 51]. These local environments also approximate many isolated catalytic metal sites, including single‐atom catalyst motifs [11, 19, 21], while excluding the additional complexity of extended supports, nanoparticles, structural disorder, and coexisting local environments [15, 17].

AI‐EXAFS accurately reproduces FEFF [22] reference spectra across chemically diverse local environments, with ablation analysis demonstrating how the graph representation, model architecture, normalization strategy, and EXAFS‐specific optimization objective contribute to predictive performance. Evaluation on molecular and supported Pt systems further shows that the model recovers the principal spectral characteristics when complete local environments are not directly represented in training. More broadly, AI‐EXAFS establishes a scalable structure‐to‐spectrum framework in a well‐defined molecular domain, enabling direct inference with minimal per‐structure setup and efficient batched evaluation of candidate structures, while providing a methodological foundation for progressively extending data‐driven EXAFS prediction to the structural heterogeneity, disorder, and ensemble complexity encountered in experimental systems.

## 2 Encoding EXAFS Physics in the Graph Representation

Establishing a direct mapping between atomic structure and the EXAFS signal requires a graph representation that reflects the physical origin of the spectrum. In XAS, absorption of an X‐ray photon by a core‐level electron generates a photoelectron that propagates away from the absorbing atom and is scattered by neighboring atoms [12]. In the near‐edge XANES region, the lower‐energy photoelectron undergoes strong multiple scattering, giving rise to highly geometry‐sensitive spectral fingerprints. In the higher‐energy EXAFS region considered here, the measured oscillatory signal, χ(k), is dominated by backscattering from neighboring atoms and arises from interference between the outgoing photoelectron wave and waves scattered back to the absorber along single‐ and multiple‐scattering pathways (Figure 1a). Single‐scattering contributions are primarily determined by absorber–scatterer distances and the chemical identity of the scattering atoms, which influence the phase and amplitude of oscillations. Multiple‐scattering pathways additionally depend on how neighboring atoms are arranged around the absorber, including local connectivity, bond angles, path topology, and sequential scattering interactions. The structural information underlying the EXAFS response therefore extends from the identity and distance of individual scatterers to the broader organization of atoms around the absorbing center [12, 13, 14].

**FIGURE 1:**
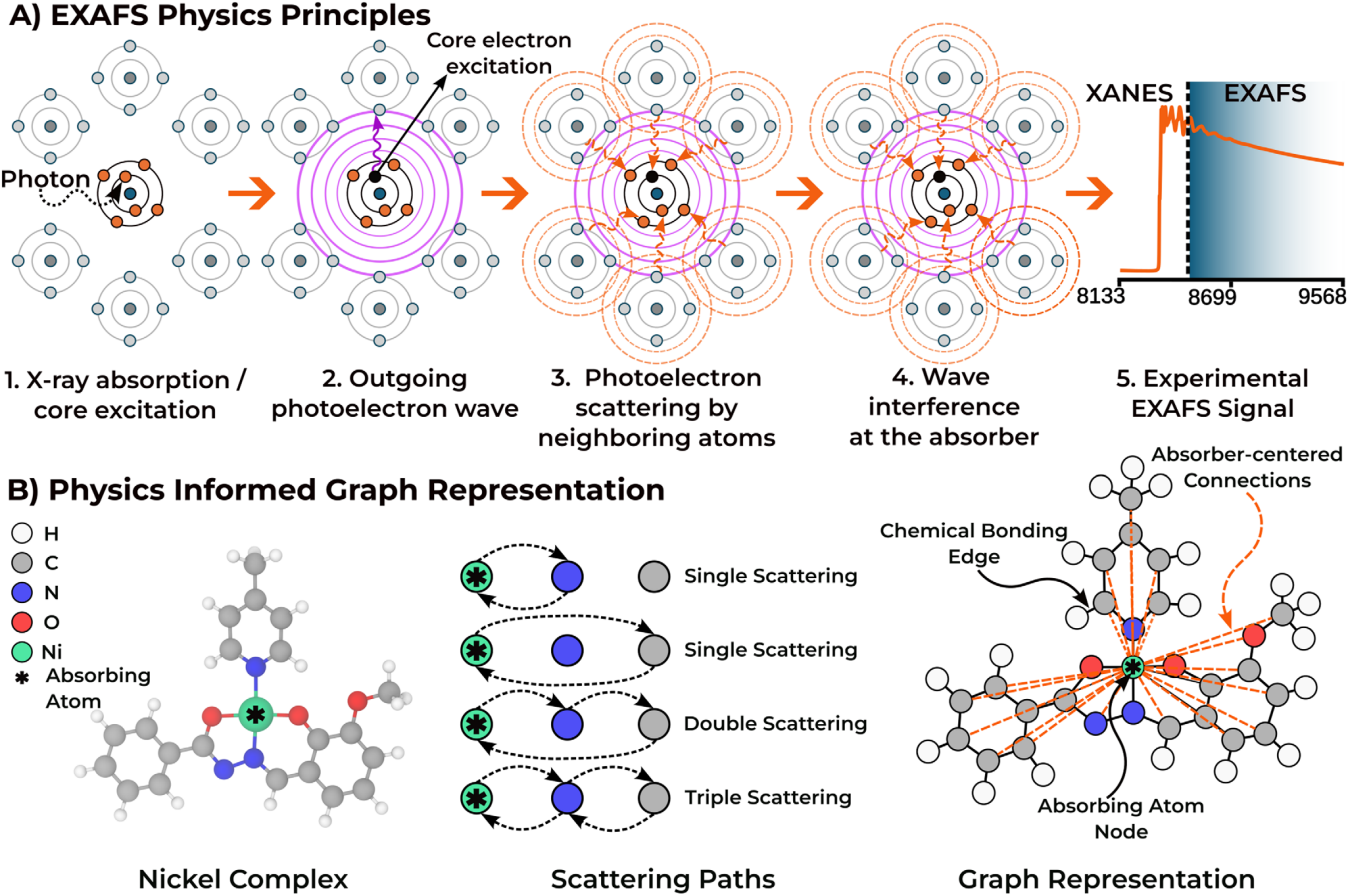
Physics‐informed representation of EXAFS scattering. (A) Schematic illustration of EXAFS generation through propagation and scattering of the photoelectron wave within the local atomic environment surrounding the absorber. (B) Absorber‐centered graph representation used in AI‐EXAFS, illustrating the atomic and geometric information provided to the model.

To represent these complementary levels of structural information, AI‐EXAFS represents each structure as an absorber‐centered graph that combines local atomic connectivity with direct connectivity to the absorber. Nodes correspond to atoms and encode atomic identity. The edge set contains two complementary components: bond‐like local connections between neighboring atoms, determined through Voronoi tessellation and covalent‐radius criteria, and absorber‐centered connections linking the absorbing atom to every other atom in the structure (Figure 1b and Note S1). The absorbing atom is connected to all atoms in the structure, independently of whether they are chemically bonded, ensuring that every potential scatterer is directly related to the absorbing center. The local connections preserve how atoms are organized relative to one another. Interatomic distances are further included as edge attributes, with each edge carrying the corresponding scalar interatomic distance. Importantly, these graph edges do not explicitly encode physical scattering paths, their ordered scattering sequences, or associated path geometry and properties. Together, the absorber‐centered and local connections, their interatomic distances, and the atomic identities provide a structural description that captures both the pairwise relationships to potential scatterers and the local organization in which they are embedded. The model can therefore learn scattering‐relevant structure‐to‐spectrum relationships from this absorber‐centered structural context without requiring physical scattering paths to be specified explicitly [22].

## 3 Dataset Construction

Establishing a direct structure–spectrum mapping requires a consistent dataset of atomic structures and corresponding EXAFS signals. To this end, we constructed a systematically defined dataset enabling controlled exploration of how local atomic environments give rise to EXAFS features across diverse transition‐metal coordination environments.

The dataset comprises 85,991 mononuclear transition‐metal complexes from the tmQM_wB97MV database [50, 51], together with their corresponding theoretical (FEFF‐generated) EXAFS spectra. The structures span transition metals from groups 3–12 (Figure 2a) and cover a broad range of ligand classes, coordination numbers, bond distances, and local geometries. Although molecular, the structures provide a broad and well‐defined library of transition‐metal coordination environments. This is a relevant starting point for EXAFS because the technique is primarily sensitive to local absorber‐centered structure, including absorber–scatterer distances, scattering‐atom identity, coordination geometry, and the organization of surrounding atoms relevant to the EXAFS response. The dataset thus offers a controlled theoretical reference space for establishing the fundamental mapping between well‐defined three‐dimensional atomic environments and their corresponding theoretical EXAFS spectra [21].

**FIGURE 2:**
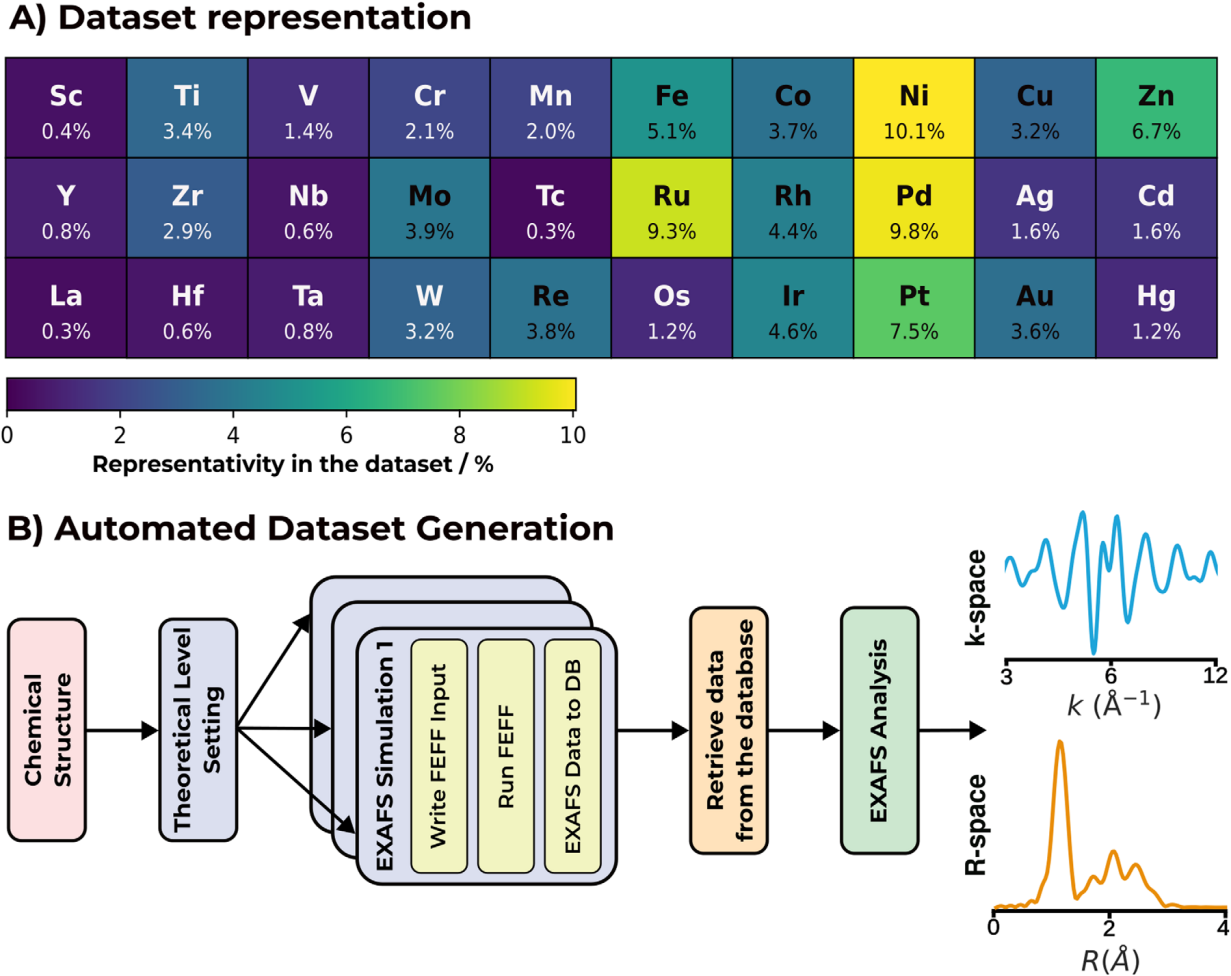
Dataset composition and automated EXAFS generation workflow. (A) Distribution of absorbing elements in the tmQM_wB97MV dataset [50, 51]. (B) Schematic workflow illustrating the generation of theoretical EXAFS data from atomic structures and their processing into consistent structure–spectrum pairs for AI‐EXAFS.

The dataset should therefore be viewed as a controlled theoretical reference space for learning structure–spectrum relationships, rather than as a direct representation of full experimental EXAFS complexity. It does not explicitly include thermal or static disorder, dynamic structural fluctuations, mixtures of species, ensemble averaging, or experimental noise. This controlled construction is intentional: it allows the model to first learn how defined three‐dimensional atomic structures map onto EXAFS responses under consistent simulation and processing conditions. Establishing this baseline also provides a basis for extending the framework along complementary directions. Structurally, the training domain can be expanded toward more heterogeneous environments, including supported single‐atom catalysts, surface species, and disordered solids. At the spectral level, future training data can incorporate these additional physical effects, progressively connecting structure‐to‐spectrum learning with the additional complexity of experimental EXAFS.

To generate the corresponding EXAFS spectrum for each structure, we implemented an automated and standardized simulation workflow. All FEFF and spectral‐processing parameters were fixed according to established EXAFS protocols, ensuring that differences between spectra arise from structural variation rather than from user‐dependent choices in path selection, normalization, background subtraction, or Fourier transformation (Note S2, S3, and Table S1–S3). The calculated spectra were then processed using a consistent Larch [52] procedure to obtain consistent k‐ and R‐space representations (Figure S1 and Tables S4 and S5). The complete workflow produces a unified structure–spectrum dataset in which each atomic structure is paired with a consistently generated and processed theoretical EXAFS response (Figure 2b).

## 4 Model Architecture for Structure–Spectrum Prediction

To learn the mapping between three‐dimensional atomic structure and EXAFS spectra, AI‐EXAFS employs a graph neural network that takes an absorber‐centered atomic environment as input and predicts the corresponding EXAFS signal directly in k‐space. Building on the graph representation defined above, the network learns a direct structure‐to‐spectrum mapping from the encoded atomic environment. The network predicts $\chi \left(k\right)\cdot k^{2}$ directly, while R‐space spectra are obtained afterward by Fourier transformation and are used only for evaluation and visualization.

The architecture follows the absorber‐centered graph representation introduced above (Figure 3, Note S5). Atomic features, including absorber and scatterer elements, are encoded as node features and embedded into a latent space, and subsequently refined through residual layers and graph convolutions that propagate information across the graph edges. Through this message‐passing process, each atomic representation is updated using information from connected atoms and the corresponding distance attributes [53]. Repeated propagation progressively integrates this information across the graph, allowing structural relationships involving multiple atoms to be represented within the learned node features. In this way, scattering‐relevant structure–spectrum relationships can be learned without explicitly enumerating FEFF‐like scattering paths.

**FIGURE 3:**
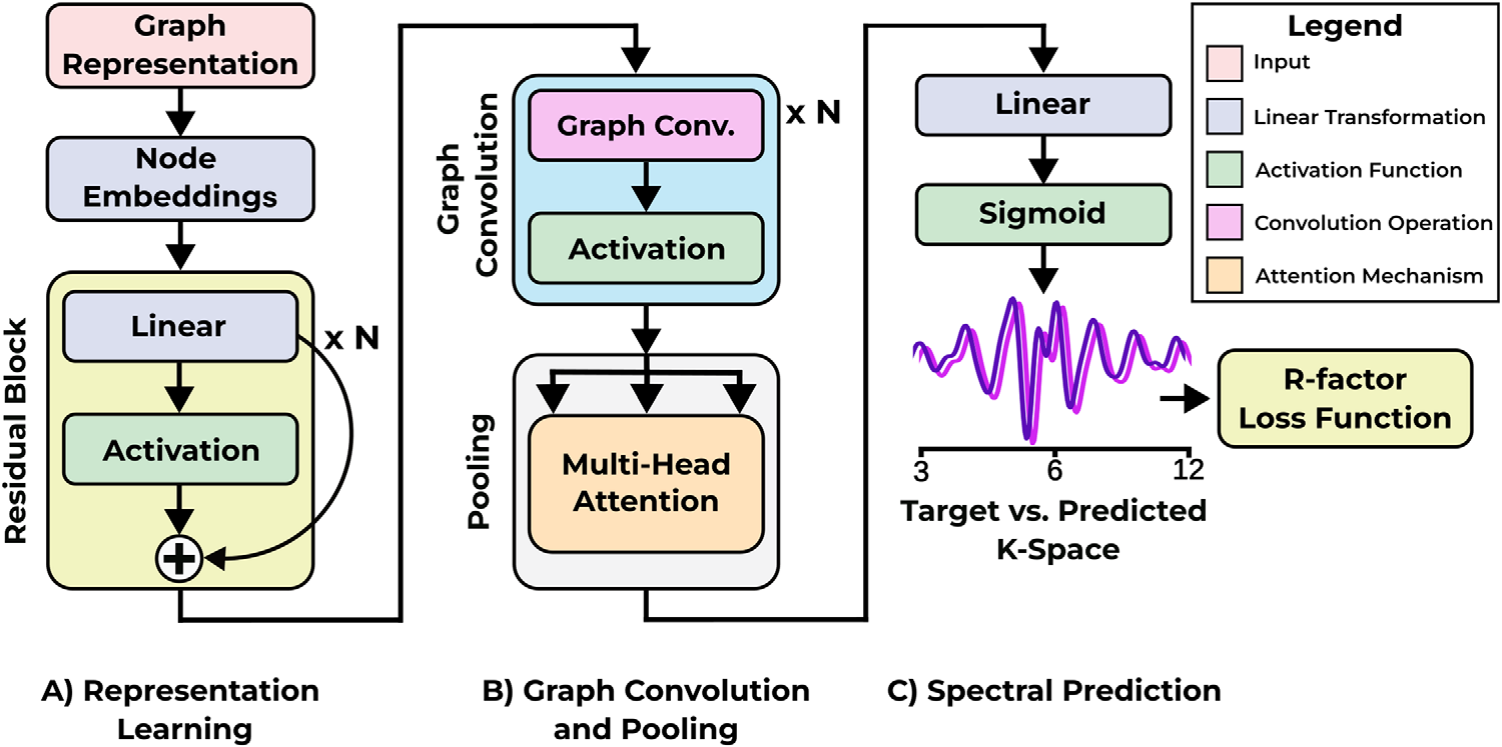
AI‐EXAFS architecture for end‐to‐end prediction of EXAFS spectra. (A) Representation learning: absorber‐centered graph representation of the local atomic environment used as model input. (B) Graph convolution and pooling: message passing progressively integrates structural information across the graph, followed by attention pooling into a global representation. (C) Spectral prediction: prediction of the $\chi \left(k\right)\cdot k^{2}$ spectrum and optimization through an EXAFS *R*‐factor loss in k‐space.

A multi‐head attention pooling stage [54, 55] then aggregates the learned atomic representations into a global representation through learned attention weights. To enable learning across different absorbing elements, spectra are normalized separately for each element using element‐specific scaling parameters derived exclusively from the training data (Note S4). This preserves relative amplitude variations within a given absorber, while enabling chemically distinct systems to be included in a single training framework. The resulting global representation is finally projected onto the predicted $\chi \left(k\right)\cdot k^{2}$ profile through a sigmoid activation. The normalized training targets occupy the interval [0.1,0.9], reserving headroom within the bounded output range for amplitudes extending beyond the element‐specific training extrema. The same training‐derived normalization parameters are used to recover the original spectral scale prior to further analysis.

Model training is guided by an EXAFS‐specific objective consistent with established EXAFS practice. A differentiable formulation of the *R*‐factor is used to penalize differences between the predicted and reference spectra, aligning optimization with an established measure of EXAFS spectral agreement (Table S6).

## 5 Model Performance

We first evaluate AI‐EXAFS through an in‐distribution benchmark within the same theoretical tmQM_wB97MV domain used for training. Evaluation was performed on the held‐out test subset generated by the fixed pseudo‐random split, comprising transition‐metal complexes spanning groups 3–12 and a broad range of coordination environments. Within this domain, the full scattering‐based model accurately reproduces the FEFF‐generated $\chi \left(k\right)\cdot k^{2}$ reference spectra, capturing both oscillation phase and relative amplitude variations across the k‐range. Prediction error was quantified using the conventional EXAFS *R*‐factor as a spectral‐error metric [56, 57]. The distribution of prediction errors is strongly concentrated at low *R*‐factor values, with average *R*‐factors of 0.04 in k‐space and 0.02 in R‐space (Figure S2). Most predictions fall below approximately 0.05 in k‐space and 0.02 in R‐space.

Representative predictions spanning all performance quartiles further show that AI‐EXAFS captures the main spectral features across chemically diverse absorbing elements and coordination environments (Figure 4). Even for lower‐performance examples within the fourth quartile, the model preserves the dominant oscillatory behavior in k‐space and the principal coordination‐shell features in R‐space. This suggests that prediction errors arise primarily from local deviations in amplitude and fine spectral details rather than failure to recover the underlying scattering response (Figures S3 and S4).

**FIGURE 4:**
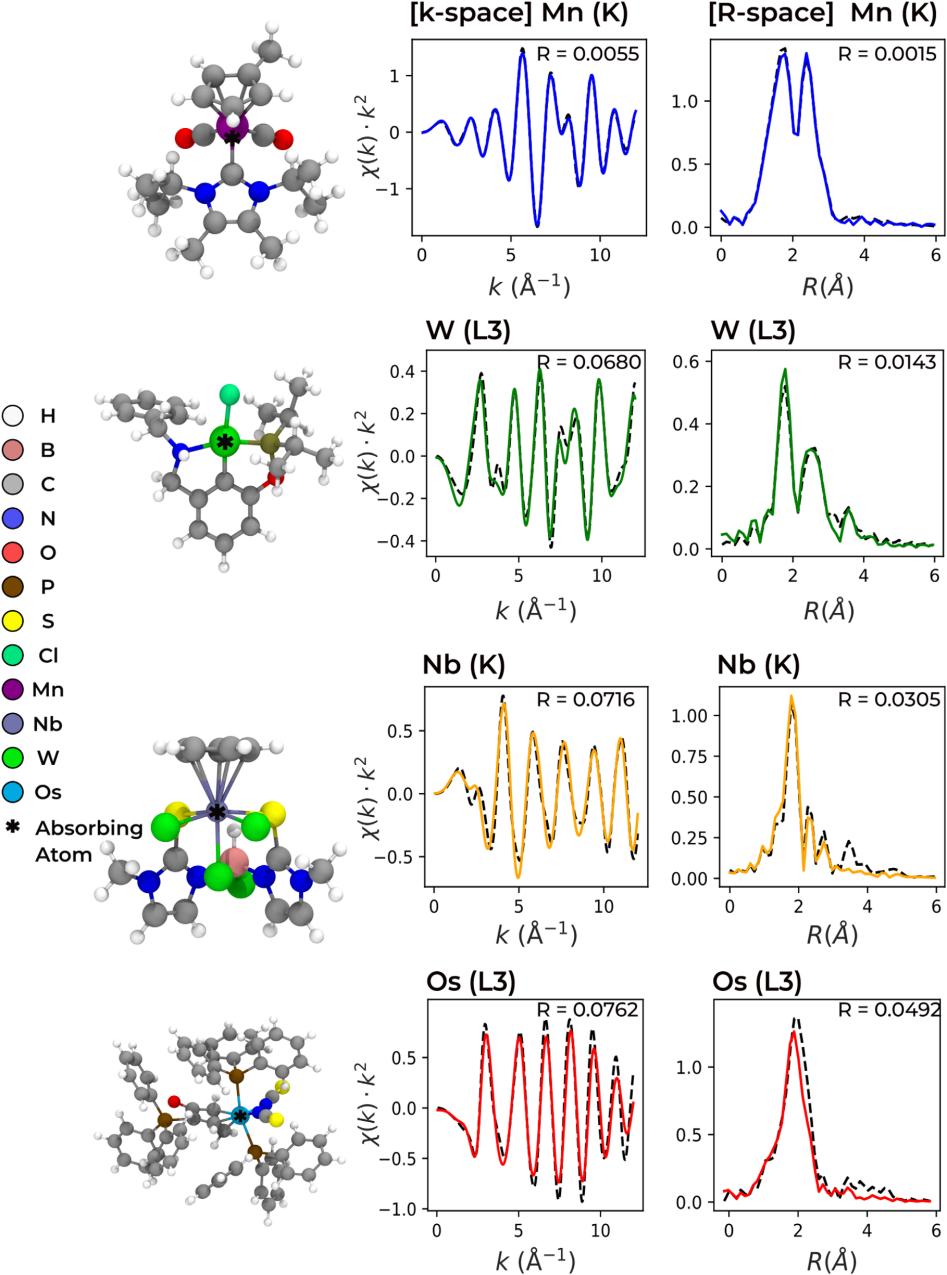
Representative AI‐EXAFS predictions across different coordination environments. Comparison between FEFF reference (dashed black) and AI‐predicted (solid colored) EXAFS spectra in k‐space for representative systems spanning different absorbing elements and local coordination environments. Corresponding Fourier‐transformed spectra in R‐space are shown on the right. Colors indicate prediction quartiles ranked by *R*‐factor performance, with blue denoting lower‐error predictions and red denoting higher‐error predictions; see Figure S2.

The best performance is observed for mid‐series 3d and 4d transition metals such as Cr, Mn, Fe, Co, Ru, Rh, and Pd, where average k‐space *R*‐factors remain close to or below ∼0.02–0.04. However, this trend is not determined by dataset representation alone, as sparsely represented elements such as Tc and Os, each accounting for less than 1.5% of the dataset, still achieve reasonable k‐space *R*‐factors of 0.0467 and 0.0464, respectively. In contrast, larger deviations are observed for some underrepresented elements, particularly the group‐3 absorbers Sc (0.1292), Y (0.2340), and La (0.2787), as well as Hf (0.0809), Ta (0.1042), and Hg (0.0947) (Figures S5 and S6). Across all elements, R‐space errors remain systematically lower than the corresponding k‐space values, by approximately a factor of two, demonstrating robust recovery of the dominant coordination‐shell structure despite variations in elemental representation and chemical diversity (Figure S5).

## 6 Contributions to Accurate EXAFS Prediction

To identify which aspects of the physics‐informed framework contribute to its predictive performance, we performed a systematic ablation analysis in which components of the representation, architecture, and training procedure were selectively removed from the full model and the resulting degradation in performance was evaluated (Note S6, Table S7 and Figure S7). Such analyses are important because high predictive accuracy alone does not reveal which methodological choices are most important for learning the structure–spectrum relationship. By progressively simplifying the framework, we can determine how the structural information provided to the model, its propagation and aggregation through the network, and the training objective each contribute to predictive accuracy. To complement the statistical analysis, two representative systems corresponding to the average model behavior were additionally examined qualitatively in both k‐ and R‐space (Figure 5, Figures S8 and S9).

**FIGURE 5:**
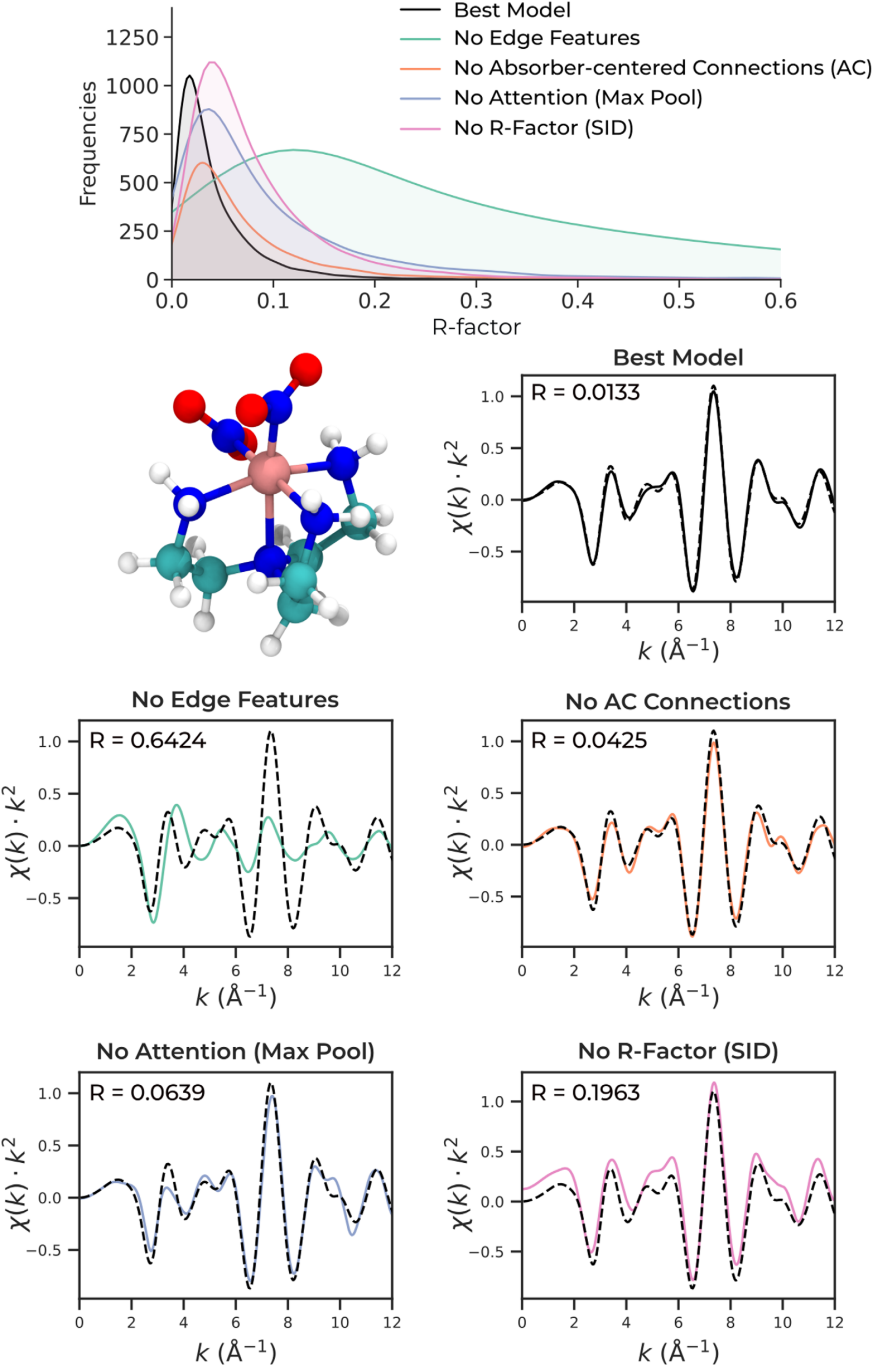
Representative ablation analysis of Co K‐edge EXAFS predictions. *R*‐factor distributions for the full AI‐EXAFS model and representative ablated variants, together with predicted (solid colored) and reference (dashed black) $\chi \left(k\right)\cdot k^{2}$ spectra for a representative Co complex, with the reported *R*‐factors specific to this structure (see Table S7 for average values). The selected ablations probe the contributions of distance information, absorber‐centered connectivity, pooling strategy, and training objective to spectral prediction.

Removing interatomic distance information by eliminating edge features leads to a substantial increase in error, with the mean *R*‐factor increasing by approximately 812% (Figure S7). Although the predicted spectra retain the overall oscillatory shape, they exhibit clear phase distortions (Figure 5) and reduced sensitivity to small bond‐length variations (Figure S8). This behavior is consistent with the EXAFS equation, where the effective scattering distance enters the oscillatory phase term, 2kR+ϕ(k), and shows that explicit distance information is critical for resolving the spectral variations associated with changes in atomic separation.

Removing explicit connectivity between the absorber and neighboring atoms results in a further performance degradation of approximately 71% (Table S7). This ablation tests the role of providing direct graph connections between the absorber and potential scatterers, independently of the local bond‐like connectivity that remains in the graph. The resulting spectra exhibit missing or shifted features associated with the first coordination shell (Figures S8 and S9), highlighting the importance of absorber‐centered connectivity for making absorber–scatterer relationships directly accessible during message passing.

At the architectural level, removing neural network residual connections in the message‐passing network leads to a clear reduction in accuracy (mean *R*‐factor of approximately 0.05, Table S7), showing that retaining and refining learned atomic representations across successive network layers contributes to spectral prediction. Replacing the attention‐based pooling with fixed aggregation increases the mean *R*‐factor from 0.043 to 0.048–0.102 depending on the pooling scheme (Table S7). This comparison probes how the atomic representations generated by message passing are combined into the global representation used for spectral prediction. The max‐pool variant produces spectra that reproduce the dominant oscillation frequencies but fail to accurately capture relative amplitude differences between coordination environments in the representative predictions (Figure 5 and Figure S9), indicating that learned attention‐based aggregation provides a more effective combination of the available atomic representations than fixed pooling schemes.

Removing the sigmoid activation at the output layer further reduces performance (mean *R*‐factor of ∼0.06, Table S7) and produces non‐finite losses during optimization. This ablation tests the role of matching the model output to the bounded element‐specific normalization scheme; its degradation indicates that reserving a defined output range contributes to stable optimization and prediction within the normalized spectral domain.

Finally, replacing the EXAFS *R*‐factor loss with alternative regression objectives degrades predictive performance. The MAE objective increases the average *R*‐factor from 0.043 to 0.049, while the spectral information divergence (SID) objective increases it to 0.084 (Table S7), with distortions in oscillation phase and relative spectral amplitudes observed in the representative predictions. These comparisons directly test how the optimization criterion prioritizes deviations across the predicted spectrum. This indicates that the choice of optimization objective influences how spectral deviations are prioritized during training, with the EXAFS *R*‐factor providing a loss formulation more consistent with conventional EXAFS spectral comparison.

Together, these results show that AI‐EXAFS performs well not simply because of neural‐network flexibility, but because complementary choices at the representation, architecture, output, and optimization levels each make measurable contributions to predictive performance.

## 7 AI‐EXAFS in Experimentally Relevant Pt Environments

To examine AI‐EXAFS beyond the theoretical tmQM_wB97MV test set, AI‐EXAFS was applied to two experimentally characterized Pt systems. A molecular Pt complex and a Pt single‐atom catalyst were selected to probe progressively more complex Pt–Cl environments, from a well‐defined molecular coordination sphere to a supported metal site containing structural features absent from the molecular training domain. Both systems contain Pt–Cl coordination represented within the training data, although their complete structural environments are not reproduced by individual training examples. Their relationship to the training distribution was therefore explicitly assessed using Smooth Overlap of Atomic Positions (SOAP)‐based structural similarity analysis and retraining after removal of highly similar environments (Note S8 and Figures S10–S12). The present implementation predicts EXAFS spectra from individual static structures and is not intended to perform quantitative refinement or unique structural assignment from experimental data.

We first examined the Pt $L_{3}$‐edge EXAFS spectrum of $K_{2}{\mathrm{PtCl}}_{6}$, a molecular reference featuring a well‐defined ${\mathrm{PtCl}}_{6}$ coordination (Figure 6a). Although Pt–Cl distances are broadly sampled during training, no Pt structure contains six first‐shell chloride donors, and the complete ${\mathrm{PtCl}}_{6}$ coordination motif is absent from the dataset (Figure S10). AI‐EXAFS closely reproduces the principal oscillatory and coordination‐shell features of the corresponding FEFF spectrum, with the predicted and experimental spectra sharing these principal features in k‐ and R‐space. When the model is retrained after removing structures with SOAP similarity greater than or equal to 0.80, quantitative agreement with FEFF decreases while these principal spectral characteristics are retained (Figure S11). This sensitivity indicates that structurally similar training environments contribute to the prediction for this molecular reference.

**FIGURE 6:**
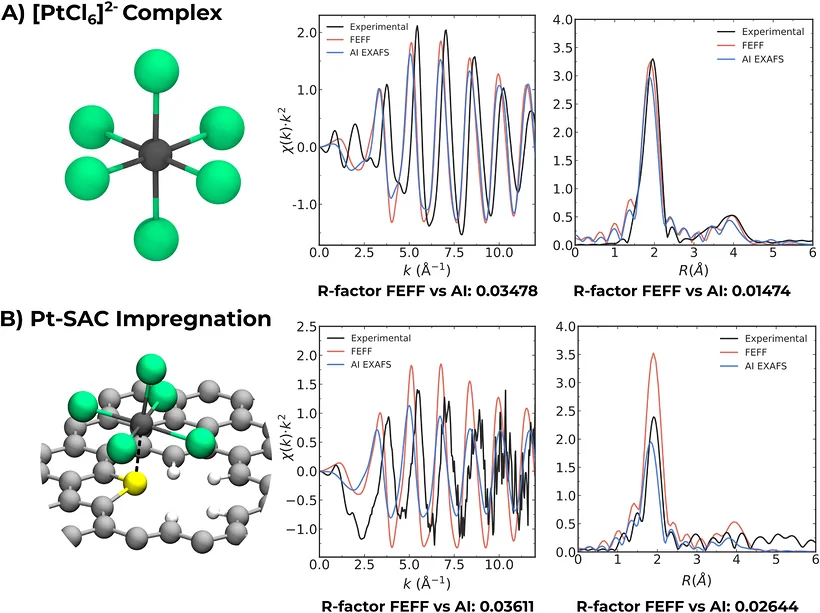
Comparison of AI‐EXAFS with experimental and FEFF Pt $L_{3}$‐edge spectra. (A) Octahedral ${\left[{\mathrm{PtCl}}_{6}\right]}^{2-}$ complex and (B) supported Pt single‐atom catalyst, shown with the corresponding experimental spectra, FEFF simulations, and AI‐EXAFS predictions in k‐ and R‐space. Black, red, and blue curves denote experiment, FEFF, and AI‐EXAFS, respectively. Predictions were obtained with the model trained on the 68,791‐structure training subset; reported *R*‐factors compare AI‐EXAFS with FEFF. Experimental spectra are included for qualitative comparison, while the SOAP‐based retraining stress test is reported in Note S8 and Figures S11 and S12. Experimental data were obtained from the XAS Database for panel (A) and from Ref. [59] for panel (B).

We next considered a Pt single‐atom catalyst prepared by impregnation of activated carbon with a chloroplatinic acid precursor followed by mild thermal treatment [58, 59]. This system was selected because its synthesis and structure have been extensively investigated experimentally, providing a chemically well‐grounded basis for constructing representative structural models. Previous XPS and EXAFS studies have shown that freshly prepared catalysts contain atomically dispersed Pt species coordinated by multiple chlorine ligands, with highly chlorinated Pt environments constituting important structural motifs under mild preparation conditions [58].

Based on the highly chlorinated Pt environment observed experimentally, we considered a ${\mathrm{PtCl}}_{5}$ configuration directly coordinated to the carbon support through S, consistent with support‐binding motifs explored through DFT [59] (Figure 6b). This ${\mathrm{Cl}}_{5}S$ first‐shell composition occurs in only one training structure, while the extended graphitic carbon support has no counterpart among the discrete molecular complexes used for training (Note S8). AI‐EXAFS reproduces the principal features of the corresponding FEFF spectrum, while also retaining the main spectral characteristics observed experimentally. Removing the most similar training environments produces only minor changes in the prediction (Figure S12).

These comparisons serve complementary purposes: FEFF provides the quantitative reference for evaluating AI‐EXAFS, whereas experiment places the predicted spectra in the context of the principal features observed in real systems. Because the present framework describes individual static configurations, effects arising from thermal or dynamic disorder, ensemble averaging, and coexisting local environments remain outside its scope. Differences in agreement with experiment are therefore interpreted only within this qualitative context and not as evidence that AI‐EXAFS recovers physical effects absent from its training targets.

More broadly, this comparison highlights a challenge that extends beyond the particular spectral model employed. In single‐atom catalysts, the experimentally relevant structure is often not a unique atomic configuration but a population of related coordination environments that may evolve during synthesis, activation, and operation [59]. Consequently, agreement between a calculated and an experimental EXAFS spectrum cannot by itself establish a unique structural assignment. Reliable interpretation requires combining spectroscopic information with independent knowledge of catalyst synthesis, chemistry, and likely catalyst speciation [60]. In this context, AI‐EXAFS should be viewed as a forward modeling tool that can rapidly evaluate chemically plausible structural hypotheses rather than as a standalone route to catalyst structure determination.

## 8 Conclusions

This work introduces AI‐EXAFS, a physics‐informed machine‐learning framework that predicts EXAFS spectra directly from three‐dimensional atomic structures across chemically diverse transition‐metal systems. By combining absorber‐centered graph representations with a message‐passing architecture and an EXAFS‐specific optimization objective, the model learns direct relationships between local atomic geometry and the corresponding $\chi \left(k\right)\cdot k^{2}$ response.

A central outcome of this work is that predictive performance depends on complementary choices in how atomic structure is represented and processed by the model. The ablation analysis shows that absorber‐centered connectivity and interatomic distances provide key structural information, while message passing, attention‐based aggregation, and the EXAFS‐specific objective determine how this information is combined for spectral prediction. These components enable the model to reproduce spectral variations associated with local coordination and higher‐order scattering‐relevant geometry without explicitly enumerating FEFF‐like scattering paths.

Within the theoretical reference domain, AI‐EXAFS accurately reproduces FEFF‐generated spectra across diverse transition‐metal coordination environments. Evaluation on two experimentally characterized Pt systems extends this assessment to local environments not fully represented by individual structures in the molecular training set. AI‐EXAFS retains their principal spectral characteristics, while the different effects of removing closely related training environments highlight the importance of structural coverage for predictive accuracy. These results support the use of AI‐EXAFS as a scalable forward model for direct structure‐to‐spectrum screening across large sets of candidate structures.

At the same time, the present implementation should be viewed as a foundation rather than a complete solution to experimental EXAFS interpretation. The model is trained on theoretical spectra generated from single static molecular structures and does not yet explicitly account for Debye–Waller damping, thermal or dynamic structural disorder, mixtures of species, ensemble averaging, or experimental noise. Extending the training domain to thermally sampled configurations, more heterogeneous atomic environments, and experimentally validated spectra would provide a direct route to incorporating these additional sources of structural and spectral complexity.

Within this scope, AI‐EXAFS provides a structure‐to‐spectrum forward model that can be integrated with existing experimental workflows to evaluate candidate structures against measured spectra or pure spectral components obtained from decomposition of evolving datasets. Rather than replacing conventional EXAFS analysis, such a workflow could use direct inference with minimal per‐structure setup and efficient batched evaluation to prioritize physically plausible local structures for subsequent fitting and refinement. This establishes a practical pathway from the present molecular model toward more systematic structure‐resolved EXAFS analysis as the training domain is expanded to encompass the structural and spectral complexity of experimental systems.

## Conflicts of Interest

The authors declare no conflicts of interest.

## Supporting information


**Supporting File**: advs78226‐sup‐0001‐SuppMat.pdf.

## Data Availability

The data that support the findings of this study are openly available in ChemrXiv at (https://doi.org/10.26434/chemrxiv‐2026‐sv3f6). The AI‐EXAFS framework developed in this study is openly available on GitHub at (https://github.com/LopezGroup‐ICIQ/ai‐exafs), including source code, training workflows, model architectures, and documentation for reproducible training and inference (Note S7). A public web‐based implementation is additionally accessible at (https://ai‐exafs.iciq.es/) ‐ (https://doi.org/10.19061/ai‐exafs), enabling browser‐based EXAFS prediction directly from uploaded atomic structures together with interactive visualization of the corresponding k‐ and R‐space spectra (Note S9). All datasets used in this work, including processed graph representations, synthetic EXAFS spectra, training and validation splits, model checkpoints, and supplementary analysis outputs, are archived on Zenodo at (10.5281/zenodo.22231932). The repository further contains scripts and metadata required to reproduce the reported results and figures, together with the exact k‐ and R‐space grids provided in both NumPy (.npy) and plain‐text (.csv) formats.
