# Supplementary material for: AI‐EXAFS: Physics‐Informed Prediction of EXAFS from Atomic Structure
Source: Adv Sci (Weinh). 2026 Oct 18:e78226. Online ahead of print. doi: 10.1002/advs.78226 (PMC13676989; doi:10.1002/advs.78226)
Supplement: Supplementary file 1 [file ADVS-9999-e78226-s001.pdf]

Supporting Information: AI-EXAFS: Physics-Informed Prediction of EXAFS from Atomic Structure

Javier Heras-Domingo,[†,‡,⊥] Andrea Ruiz-Ferrando,[†,¶,§,⊥] Adam H. Clark,[‖,§] Stephan Pollitt,[‖,§] Sharon Mitchell,[¶,§] Javier Pérez-Ramírez,[¶,§] and Núria López[*,†]

†*Institute of Chemical Research of Catalonia (ICIQ-CERCA), The Barcelona Institute of Science and Technology (BIST), Avinguda Països Catalans 16, Tarragona 43007, Spain*

‡*Departament de Química Inorgànica i Orgànica (Secció de Química Inorgànica), Institut de Química Teòrica i Computacional (IQTC), Universitat de Barcelona, C/ Martí i Franquès, 1, Barcelona, 08028, Spain.*

¶*Institute for Chemical and Bioengineering, Department of Chemistry and Applied Biosciences, ETH Zurich, Vladimir-Prelog-Weg 1, 8093 Zurich, Switzerland*

§*NCCR Catalysis, 8093 Zürich, Switzerland*

‖*Paul Scherrer Institute, CH-5232 Villigen, Switzerland*

⊥*These authors contributed equally to this work.*

E-mail: nlopez@iciq.es

Contents

S1 Graph Representation **S-3**

S2 Conventional EXAFS workflow and Relation to AI-EXAFS **S-5**

S3 EXAFS Simulation and Processing Workflow **S-6**

S4 Element-resolved Normalization of EXAFS Spectra **S-7**

S5 Model Architecture, Training, and Optimization **S-9**

S6 Ablation Study: Methodology and Evaluation Protocol **S-11**

S7 Implementation Details **S-13**

S8 Representation of Pt–Cl Environments in the Training Set **S-14**

S9 Web-app Application **S-16**

References **S-37**

S1. Graph Representation

Each molecular entry was encoded as an absorber-centered graph in which the absorbing atom corresponded to the transition-metal center probed by EXAFS. The input pymatgen[S1] `Structure` was converted into an ASE object[S2] and translated such that the absorbing atom occupied the Cartesian origin. Atomic coordinates were therefore expressed relative to the absorber, consistent with the local and element-specific nature of the EXAFS response.

Atoms were represented as graph nodes initialized through one-hot encodings of the atomic number using $\text{Max}_Z = 92$. Atomic Cartesian coordinates were retained as positional information defining absorber–scatterer distances and local scattering geometries relevant to the EXAFS signal.

Edges were constructed as the union of bond-like local connectivity and explicit absorber-centered connectivity. Local connectivity was determined from candidate atom pairs generated through Voronoi tessellation[S3] of the atomic coordinates. A candidate pair (i, j) was retained when the interatomic distance was smaller than the sum of the corresponding covalent radii plus a tolerance of 0.5 Å. For pairs involving the absorber, an additional slack factor ($sf = 1.5$) was introduced to ensure inclusion of the first coordination shell. Absorber-centered connectivity was then added by connecting the absorbing atom to all remaining atoms in the structure. The resulting edge sets were concatenated, deduplicated, and symmetrized to generate an undirected graph representation.

Each edge was initialized with the interatomic distance as a scalar edge attribute with shape $(n_{\text{edges}}, 1)$, where n_{edges} denotes the number of directed edges after symmetrization. The graph therefore explicitly provides the model with atomic identities, connectivity, and scalar interatomic distances. Disconnected graphs, if encountered, were reduced to their largest connected component and retained only when this component contained the absorbing transition-metal atom. This functionality was implemented as a preprocessing safeguard; however, no structure in the present dataset triggered exclusion on this basis.

The target associated with each graph was the k^2-weighted EXAFS signal, $k^2\chi(k)$. Spectra were truncated at $k_{\max} = 12.0\,\text{Å}^{-1}$. Subsequently, both the corresponding k-axis and the $k^2\chi(k)$ values were resampled using one-dimensional linear interpolation onto a common fixed grid containing 928 points. This interpolation changes the number of sampling points used to represent each spectrum while retaining its overall spectral profile. The resulting spectra were retained in their original physical units during dataset construction. After the train/validation/test split was defined, element-specific normalization parameters were estimated exclusively from the training subset and applied on the fly using the procedure described in **Section S4**.

Each processed structure was stored as a PyTorch Geometric `Data` object[S4] containing node features (`x`), graph connectivity (`edge_index`), edge attributes (`edge_attr`), atomic positions (`pos`), atom metadata,

structure identifiers, and the interpolated target spectrum in its original physical units.

The complete dataset was serialized into an LMDB database together with metadata describing dataset size and filtered structures. Element-specific normalization parameters were not stored in the LMDB but were derived from the training subset after dataset splitting. An isolated-node check was included as a preprocessing consistency safeguard; however, no structures in the present dataset were removed due to graph disconnection.

S2. Conventional EXAFS workflow and Relation to AI-EXAFS

Conventional EXAFS analysis transforms X-ray absorption spectra into structural information through two coupled stages: (i) spectral processing and (ii) structural fitting. During spectral processing, the measured X-ray absorption spectrum is transformed into an oscillatory EXAFS signal through normalization, background subtraction, and conversion into photoelectron momentum (k) space using established tools such as Larch,[S5] Athena (Demeter package),[S6] and WinXAS.[S7] These operations isolate the structural component of the signal while preserving the physically meaningful oscillatory response arising from photoelectron scattering. The processed EXAFS signal is subsequently fitted using theoretically generated spectra derived from structural models, where agreement between theory and experiment is evaluated through refinement of effective structural and spectral parameters describing the local atomic environment.

Theoretical EXAFS spectra are typically generated using multiple-scattering approaches, most commonly implemented through FEFF,[S8] which computes the scattering amplitudes, phase shifts, and interference effects arising from the local atomic environment of the absorber. During structural fitting, this structure–spectrum relationship is repeatedly evaluated across different local coordination models and fitting parameterizations in order to identify descriptions consistent with experiment.

The present work focuses specifically on learning this structure–spectrum relationship from theoretically generated EXAFS spectra. Because AI-EXAFS operates directly on processed EXAFS signals, the theoretical spectra used for training and the experimental spectra included for qualitative comparison are represented within a consistent EXAFS framework (**Figure S1**). Accordingly, AI-EXAFS adopts a unified workflow for theoretical EXAFS generation and spectral processing to construct a consistent structure–spectrum dataset for learning the direct mapping between atomic structure and EXAFS response.

S3. EXAFS Simulation and Processing Workflow

Theoretical EXAFS spectra were generated using the FEFF software package (version 9.6),[S8] which implements a real-space multiple-scattering Green's function formalism for X-ray absorption spectroscopy. Within this framework, the EXAFS signal is obtained as the coherent sum of single- and multiple-scattering contributions arising from the interaction of the photoelectron with the local atomic environment surrounding the absorber. Scattering amplitudes, phase shifts, and energy-dependent potentials are computed through self-consistent field (SCF) calculations and real-space Green's functions, which additionally account for photoelectron mean free paths, inelastic losses, and core-hole screening, yielding physically realistic spectra across diverse chemical environments.

Within AI-EXAFS, theoretical EXAFS generation and spectral processing were implemented as a unified automated workflow using the FireWorks[S9] and Atomate[S10] frameworks. FEFF input files were generated through the EXAFSSet interface in Pymatgen,[S1] ensuring standardized handling of atomic coordinates and simulation settings. Atomic structures were provided directly from the molecular geometries, with the absorbing atom automatically identified for each system. The absorption edge was assigned according to the atomic number of the absorbing element. The K-edge was used for absorbers with $Z \leq 57$, comprising the $3d$ series (Sc–Zn), the $4d$ series (Y–Cd), and La, whereas the L_3-edge was used for absorbers with $Z > 57$, comprising the $5d$ series (Hf–Hg). The dataset contains no absorbing elements with $58 \leq Z \leq 71$, making this assignment unambiguous throughout the dataset.

All simulation and spectral processing parameters were defined according to expert-guided EXAFS protocols and subsequently fixed throughout the workflow to ensure that spectral variations arise from differences in atomic structure and scattering geometry rather than inconsistencies in numerical setup or signal treatment. The complete parameterization includes EXAFS generation settings (**Table S1**), which were translated into a consistent FEFF control configuration (**Tables S2–S3**), together with normalization and background subtraction parameters (**Table S4**) and Fourier transformation settings used for structural analysis and spectral comparison (**Table S5**).

Amplitude reduction factors (S_0^2) were determined from expert-supervised fits to experimental EXAFS spectra of representative metal foils (**Table S3**), with averaged values adopted when element-specific benchmarks were unavailable. Spectral processing was implemented using Larch (2025.3.0),[S5] while FEFF calculations produced the $\chi(k)$ spectra together with scattering-path and potential files, all of which were archived to ensure full reproducibility and traceability between atomic configurations and simulated EXAFS signals. While Fourier-transformed spectra were used for structural interpretation and comparison, the AI-EXAFS learning target itself remained defined in k-space.

S4. Element-resolved Normalization of EXAFS Spectra

All spectra were first truncated at $k_{\max} = 12.0\,\text{Å}^{-1}$ and resampled by one-dimensional linear interpolation onto a common fixed k-grid, yielding consistent $\chi(k) \cdot k^2$ representations for all structures. The dataset was then divided into training, validation, and test subsets before any normalization parameters were determined.

Let $y_i^{(e)}(k_j)$ denote the interpolated EXAFS signal of structure i with absorbing element e evaluated at k-grid point k_j. For each absorbing element, the minimum and maximum values were estimated exclusively from the spectra assigned to the training subset, across all training structures and k-points. These training-derived parameters were subsequently applied unchanged to the validation and test subsets (**Equations 1–2**). Normalization was therefore performed separately for each absorbing element only after dataset splitting, preserving relative amplitude differences within each element group while preventing information from the held-out subsets from entering the preprocessing of the training targets.

$$y_{\min}^{(e)} = \min_{\substack{i \in \mathcal{T}_e \\ j}} y_i^{(e)}(k_j), \tag{1}$$

$$y_{\max}^{(e)} = \max_{\substack{i \in \mathcal{T}_e \\ j}} y_i^{(e)}(k_j), \tag{2}$$

Where $\mathcal{T}_e$ denotes the training structures associated with absorbing element e. The resulting parameters were reused unchanged during validation, testing, and inference. Because the network output is bounded by a sigmoid activation, the training extrema were mapped to the interval $[\ell, h] = [0.1, 0.9]$ rather than directly to $[0, 1]$. Each spectrum was then normalized using the corresponding element-specific limits (**Equation 3**).

$$\widetilde{y}_i^{(e)}(k_j) = \ell + \left(y_i^{(e)}(k_j) - y_{\min}^{(e)} \right) \frac{h - \ell}{y_{\max}^{(e)} - y_{\min}^{(e)}}, \tag{3}$$

This procedure places spectra corresponding to the same absorber on a common numerical scale while preserving relative amplitude differences within each element group. At the same time, it avoids imposing a single global normalization across chemically distinct absorbers, which can exhibit substantially different intrinsic $\chi(k) \cdot k^2$ ranges. Using bounds of 0.1 and 0.9 also reserves headroom at both ends of the sigmoid output, preventing the training extrema from being mapped to values that can only be reached asymptotically and allowing held-out spectra to extend moderately beyond the training range.

The available headroom relative to the element-specific training amplitude range is given by,

$$\eta_{\text{below}} = \frac{\ell}{h-\ell}, \qquad \eta_{\text{above}} = \frac{1-h}{h-\ell}, \tag{4}$$

which gives $\eta_{\text{below}} = \eta_{\text{above}} = 0.125$ for $\ell = 0.1$ and $h = 0.9$. The model can therefore represent amplitudes extending by 12.5% below and above the element-specific training range. This margin exceeds the largest excursion observed for the held-out spectra under training-only normalization, while keeping all test targets within the representable sigmoid range.

Spectra were retained in the LMDB dataset in their original physical units. After the train/validation/test split was defined, the element-specific normalization parameters were computed from the training subset immediately before training, and normalization was applied on the fly to the corresponding targets. The same training-derived parameters were reused unchanged during validation, testing, and inference. Predictions were returned to the original EXAFS scale using **Equation 5**.

$$y_i^{(e)}(k_j) = \frac{\widetilde{y}_i^{(e)}(k_j) - \ell}{h-\ell}\left(y_{\max}^{(e)} - y_{\min}^{(e)}\right) + y_{\min}^{(e)}, \tag{5}$$

The spectra were analyzed in both k-space and Fourier-transformed R-space. To this end, we adapted Larch's Fourier-transform routine using its `Group` object, ensuring compatibility of the denormalized $\chi(k) \cdot k^2$ spectra and k-grid with standard EXAFS workflows. All reported evaluation metrics were calculated after applying the inverse transformation to spectra expressed in their original physical scale and are therefore independent of the selected normalized interval.

S5. Model Architecture, Training, and Optimization

Following the theoretical EXAFS generation and spectral processing workflow described in **Section S3**, all spectra were represented in k-space as $\chi(k) \cdot k^2$ signals on a common fixed grid of 928 points. Before training, the k-axis and its corresponding $\chi(k) \cdot k^2$ values were resampled consistently onto this grid using one-dimensional linear interpolation. This procedure changes the number of sampling points used to represent each spectrum while preserving the correspondence between the spectral intensities and their k-values, as well as the overall spectral profile. The interpolated spectra were retained in the LMDB database[S11] in their original physical units. The dataset was partitioned by uniform pseudo-random sampling over complete structures using PyTorch's `random_split` with a fixed generator seed of 42. The validation and test subsets each contained 8,600 complexes, while the remaining 68,791 formed the training subset. The partition was defined before any normalization parameters were determined. Element-specific normalization parameters were subsequently estimated exclusively from the training subset immediately before optimization and applied on the fly to all targets according to the procedure described in **Section S4**.

Building on the scattering-based graph representation, AI-EXAFS maps atomic structures directly to their corresponding EXAFS spectra. Node features are first projected into a latent space through a linear embedding layer and refined using residual layers with activation and skip connections to capture complex atomic correlations. A graph convolutional block then performs message passing modulated by edge distances, enabling the model to learn structural correlations from atomic identities, interatomic distances, and graph connectivity without explicitly enumerating multiple-scattering paths. Node embeddings are subsequently aggregated using multi-head attention pooling,[S12,S13] which weights atomic contributions according to spectral relevance. The pooled representation is finally mapped through fully connected layers with a sigmoid activation, yielding EXAFS spectra in the normalized output space. The training targets occupy the interval $[0.1, 0.9]$, reserving headroom within the sigmoid range for amplitudes extending beyond the element-specific training extrema.

The model was trained to predict EXAFS spectra directly in k-space, preserving the oscillatory structural information encoded in the signal while allowing subsequent transformation to R-space when required. Training was performed on the 68,791 graph–spectrum pairs of the training subset, with the 8,600 validation pairs used for model selection and the 8,600 test pairs held out for final evaluation. Optimization employed the **Adam optimizer**[S14] together with a **ReduceLROnPlateau** scheduler (patience = 5; minimum learning rate = 1.0×10^{-6}). Training was guided using a differentiable R-factor loss[S15] (**Equations 6–7**), aligning optimization with established EXAFS analysis metrics and sensitivity to structural variations.

The R-factor is defined as,

$$R = \frac{\sum(y_{\text{calc}} - y_{\text{pred}})^2}{\sum(y_{\text{calc}})^2} \tag{6}$$

where y_{calc} denotes the theoretically calculated ground-truth spectrum and y_{pred} denotes the spectrum predicted by the model. Its square-root form was used during training:

$$R_{\text{factor}} = \sqrt{\frac{\sum(y_{\text{calc}} - y_{\text{pred}})^2}{\sum(y_{\text{calc}})^2}} \tag{7}$$

The training loss was evaluated in the normalized output space, whereas all performance metrics reported in this work were calculated after applying the inverse element-specific transformation to recover spectra in their original physical units.

Hyperparameters were optimized using the Tree-structured Parzen Estimator (TPE)[S16] implemented in Optuna,[S17] exploring both training parameters (learning rate, batch size, weight decay) and architectural parameters (embedding dimension, depth, width). A **Median Pruner** terminated underperforming trials after an initial warm-up period, with evaluations every 25 epochs. Each trial was run for up to 50 epochs, and 200 trials were performed in total.

Final parameters were selected based on validation loss with early stopping to control model selection within the defined data distribution (**Table S6**). During training, both MAE and R-factor were monitored, while final performance was evaluated on the held-out test set. Fit quality was quantified using the R-factor (**Equation 6**), where values below ∼0.02–0.05 indicate good agreement.[S15,S18]

Performance was evaluated in both k-space and R-space using the EXAFS R-factor as the primary quantitative metric (**Figure S2**). Qualitative assessment was additionally performed through direct comparison between reference and predicted spectra in k-space (**Figure S3**) and after Fourier transformation to R-space (**Figure S4**), evaluating the reproduction of physically meaningful spectral features including oscillation phase, relative amplitude modulation, peak position, peak intensity, and coordination-shell features associated with distinct scattering environments. To further assess robustness across chemically distinct scattering regimes, performance was additionally analyzed as a function of the absorbing element through element-resolved average R-factors (**Figure S5**). The same element-resolved values are additionally presented in a periodic-table layout together with the percentage of test-set structures containing each absorber (**Figure S6**), allowing prediction accuracy to be compared directly with elemental representation in the dataset.

S6. Ablation Study: Methodology and Evaluation Protocol

To quantify the contribution of individual design choices in the proposed graph neural network (GNN), we performed a systematic ablation study spanning graph representation, model architecture, and training objective (**Table S7**). Rather than constructing the framework incrementally from simple to complex components, the analysis starts from the complete physics-informed model and progressively removes specific elements. This top-down strategy was adopted because the representation, architecture, and optimization objective are strongly coupled, making isolated contributions difficult to assess through sequential model construction. By selectively removing individual components from the reference model, the ablation analysis enables the impact of each design choice on the learned structure–spectrum relationship to be evaluated independently.

For each ablation, a single component of the reference AI-EXAFS framework was modified while all remaining components, training conditions, and data splits were kept fixed. All ablated variants were retrained from scratch using the optimal hyperparameters identified during hyperparameter optimization, including learning rate, batch size, optimizer settings, and architectural dimensions. Training, validation, and test splits were identical for all variants to ensure direct comparability between models.

At the level of the graph representation, ablations were designed to evaluate the contribution of specific structural information encoded in the absorber-centered graph representation. This was performed by modifying the graph generation procedure while leaving the remaining network architecture unchanged. Variants included removing edge distance attributes ("Removed edge distances") and removing the explicit absorber-centered connections between the absorbing atom and all remaining atoms in the structure ("Removed absorber-centered connections").

At the architectural level, ablations were designed to evaluate how structural information is propagated and aggregated throughout the network. These variants modified individual architectural components within the message-passing framework, including removal of residual node refinement layers ("Removed residual block"), replacement of attention-based pooling with add, mean, or max pooling, and removal of the bounded sigmoid activation at the output layer ("Removed sigmoid output activation").

At the level of the training objective, ablations evaluated how the optimization target influences the learned spectral representation. These variants preserved the same network architecture and graph representation while replacing the EXAFS R-factor loss with alternative objectives including mean absolute error (MAE), mean squared error (MSE), and spectral information divergence (SID).

Model performance was evaluated on the held-out test set using the average EXAFS R-factor and k-space mean absolute error (MAE), with relative changes reported against the reference model (**Figure S7**).

For completeness, the optimal model was also evaluated in R-space after Fourier transformation.

To complement the quantitative analysis, representative spectra were additionally examined qualitatively in both k-space and R-space (**Figures S8–S9**). Representative examples were selected from the most densely populated region of the R-factor distribution, providing examples of typical model behavior rather than isolated best- or worst-case predictions. This analysis was used to evaluate how each ablation affects characteristic spectral features, including oscillation phase, relative amplitude modulation, peak position, and peak intensity across the EXAFS signal.

S7. Implementation Details

The model was implemented in Python using PyTorch Geometric[S4] (2.8.0) on top of PyTorch,[S19] (2.9.1) with the training workflow organized using PyTorch Lightning.[S20] PyTorch Lightning (2.6.5) was used to separate data loading, model definition, and training logic into modular components, improving code readability and reproducibility. Graph objects were constructed as PyTorch Geometric data instances and stored in an LMDB database (2.3.0),[S11] enabling efficient key-value access to preprocessed graph representations, target spectra in their original physical units, and associated metadata. Element-specific normalization parameters were instead derived exclusively from the training subset after dataset splitting and immediately before optimization, and normalization was applied on the fly. The same training-derived parameters were reused unchanged for validation, testing, and inference.

Training, validation, and test subsets were generated by uniform pseudo-random sampling over complete structures using PyTorch's `random_split` with a fixed generator seed of 42, with deterministic CuDNN settings enabled for reproducibility. Model training employed the Adam optimizer together with a learning-rate scheduler based on validation performance, early stopping, and model checkpointing. The primary optimization target was the EXAFS R-factor, while additional metrics including mean absolute error, mean squared error, spectral information divergence, and spectral information similarity were logged for analysis.

A custom evaluation callback was implemented to denormalize predicted and reference spectra using the training-derived element-specific normalization parameters, compute their Fourier transforms, and export both k-space and R-space spectra for downstream comparison. Hyperparameter optimization was performed using Optuna (4.9.0),[S17] exploring both architectural and training parameters including hidden dimensionality, number of graph convolution layers, activation function, aggregation scheme, attention-pooling configuration, learning rate, and weight decay. Configuration files were managed with Hydra (1.3.6),[S21] while experiment tracking was performed through the Weights and Biases API (0.29.0).[S22]

S8. Representation of Pt–Cl Environments in the Training Set

The representation of Pt-Cl coordination within the AI-EXAFS training set was examined for $[PtCl_6]^{2-}$ and the supported $PtCl_5/C$ single-atom catalyst model. The analysis considered the occurrence of metal–chloride interactions across the dataset, the composition and bond-length distribution of Pt coordination environments, the similarity of both systems to the closest training structures, and a retraining stress test in which the most similar environments were removed. The first coordination shell was defined throughout as the set of atoms lying within 3.0 Å of the absorbing atom, and a metal–chloride contact as an absorber–chloride pair separated by less than this distance. Thus, a hexachloride complex contributes six contacts, whereas a structure with two chloride ligands contributes two. Contacts therefore describe coordination in the underlying chemical structure independently of the graph representation used by the model, in which the absorbing atom is connected to every atom in the structure regardless of separation.

Metal–chloride coordination is broadly represented in the training set. Among the 68,791 training structures, 19,148 contain at least one first-shell chloride, corresponding to 30,364 metal–chloride contacts distributed across all 30 absorbing elements (**Figure S10a**). The Pt subset comprises 5,160 structures, of which 1,984 contain at least one Pt–Cl interaction, giving 3,430 Pt–Cl contacts with a mean distance of 2.304 Å and 90% of values between 2.227 and 2.389 Å. The mean Pt–Cl distances of $[PtCl_6]^{2-}$ and $PtCl_5/C$, 2.340 and 2.331 Å, respectively, therefore lie within the range sampled during training (**Figure S10d**).

Although the relevant Pt–Cl distances are well represented, neither system is fully represented by an equivalent structural environment in the training set. Most Pt environments contain zero to two first-shell chlorides, six contain five, and none contains six (**Figure S10c**). The same trend extends across the dataset: no structure for any absorbing element contains six chloride donors or a homoleptic halide first shell above fourfold coordination. Sixfold coordination itself is common, occurring in 10,792 training structures with other combinations of donors, while Pt–Cl interactions occur within a chemically diverse Pt subset spanning 14 first-shell donor elements and 2,185 distinct first-shell compositions (**Figure S10b**). Thus, coordination number, donor identities, and absorber–scatterer distances relevant to the $[PtCl_6]^{2-}$ environment are represented individually across the training set, but not in the specific combination defining the complete $PtCl_6$ coordination environment.

The supported catalyst presents a complementary case. Its first shell comprises five chlorides together with a sulfur atom of the support at 2.533 Å, giving a Cl_5S composition that occurs in one training structure, corresponding to a pentachlorido(sulfoxide) platinate. In this structure, the sulfur belongs to a molecular ligand and lies at 2.42 Å, while the Pt–Cl distances fall within the sampled range. All 68,791 training structures are discrete mononuclear complexes, and the extended carbon support surrounding the Pt center

therefore has no counterpart in the training distribution. The catalyst thus combines a sparsely represented first-shell composition with a distinct Pt–S geometry and an extended support environment absent from the molecular training set.

Similarity to individual training structures was quantified using Smooth Overlap of Atomic Positions (SOAP) descriptors evaluated at the absorbing atom. SOAP encodes the local neighbor density through radial and angular components and is therefore sensitive to the three-dimensional arrangement of the coordination environment rather than only to absorber–scatterer distances. Descriptors were generated using DScribe[S23] with $n_{\max} = 8$, $l_{\max} = 6$, and a Gaussian broadening of $\sigma = 0.3$. Two radial cutoffs were considered: 3.0 Å to describe the first coordination sphere and 6.0 Å to include the extended local environment. Molecular training structures were treated as non-periodic, whereas minimum-image displacement vectors were used when extracting absorber-centered environments from the periodic Pt structures considered here. SOAP vectors were normalized before calculating their kernel similarity, with values approaching one indicating increasingly similar local environments.

Finally, the dependence of the model predictions on the closest training environments was evaluated through a retraining stress test. The complete dataset of 85,991 structures was screened against both Pt environments at both SOAP cutoffs. For each structure, the maximum similarity across the two Pt environments and two cutoffs was retained. A total of 228 structures exhibited a maximum SOAP similarity greater than or equal to 0.80, all corresponding to Pt environments. These structures, including the closest environments to both Pt systems, were removed from the full dataset before partitioning into training, validation, and test subsets, and the model was retrained from scratch following the same data-splitting, normalization, and training protocol described above. The screened dataset therefore contained no structure with a SOAP similarity of 0.80 or higher to either Pt environment at either cutoff.

Figures S11 and **S12** compare predictions from the original and retrained models. Removing the closest environments reduces quantitative agreement with the FEFF reference, particularly for $[PtCl_6]^{2-}$, for which the R-factor increases from 0.035 to 0.084 in k-space and from 0.015 to 0.052 in R-space. In contrast, the $PtCl_5/C$ prediction remains largely unchanged, with the R-factor changing from 0.036 to 0.038 in k-space and from 0.026 to 0.019 in R-space. Despite the reduction in quantitative agreement for the molecular reference, the principal oscillatory behavior in k-space and the dominant spectral features in R-space are retained for both systems.

S9. Web-app Application

AI-EXAFS is deployed through a Streamlit[S24] (1.58.0) web application that wraps the pre-trained graph neural network (GNN) model for browser-based EXAFS prediction from user-supplied atomic structures. This deployment layer provides an interactive route from structure input to predicted EXAFS spectra, a step that is often omitted from scientific machine-learning workflows despite being essential for model reuse by non-specialist users. The application is designed for experimental/theoretical users who require a rapid structure-based estimate of the EXAFS fine-structure function, $\chi(k) \cdot k^2$, without run-time FEFF/path calculations, spectral fitting, or additional model implementation. Inference is performed on CPU with the AI-EXAFS GNN model, and the predicted k-space signal is converted to an R-space magnitude spectrum using Larch Fourier-transform routines.[S5]

Users can upload structure files in XYZ or CIF format, for molecular or periodic systems. The absorbing atom is detected automatically as the transition-metal element in the structure, and the coordinates are re-centered on this atom. For CIF inputs, the user selects a spherical cluster radius between 4 and 10 Å, with a default value of 6 Å. The corresponding finite cluster is then extracted from the periodic crystal, and periodic boundary conditions are removed. Molecular XYZ systems are used as uploaded. The application then constructs a graph as described in **Section S1**. The processed structure and derived graph are displayed side by side using py3Dmol[S25] (2.5.3) and a graph viewer. After graph construction, the user initiates prediction.

The AI-EXAFS model returns a 928-point $\chi(k) \cdot k^2$ spectrum on a fixed grid spanning $k = 0.05$ to $12\,\text{Å}^{-1}$. The corresponding spacing of the model output grid is approximately $0.01289\,\text{Å}^{-1}$, rather than $0.05\,\text{Å}^{-1}$. The 928-point output dimensionality is fixed by the model architecture and corresponds to the common grid used to represent the target spectra during training. The output is de-normalized using the training-derived element-specific Min–Max scaler (see **Section S4**) associated with the detected absorbing element. The scaler is defined from the element-specific minimum and maximum values of the training subset and maps the training range to $[0.1, 0.9]$. The corresponding inverse transformation returns the prediction to the original $\chi(k) \cdot k^2$ scale. The application displays interactive $\chi(k) \cdot k^2$ and $|\chi(R)|$ plots and provides downloadable CSV files for both spectra.

The R-space representation is not unique because it depends on the Fourier-transform convention applied to the predicted k-space signal. To enable comparison with a chosen experimental processing protocol, the application exposes the k-range, window taper, apodisation function, and maximum displayed R value. Before the Fourier transform, the predicted spectrum is internally resampled by Larch onto a uniform grid with a k-step of $0.05\,\text{Å}^{-1}$. This value is an internal Fourier-transform sampling parameter and does not

correspond to the spacing of the 928-point model output grid. The forward transform follows Larch[S5] (2025.3.0) conventions using `ftwindow` and `xftf_fast`: $\chi(k) \cdot k^2$ is transformed over the selected, windowed k-range with $N_{\text{fft}} = 2048$.

The selectable controls are k-range = 3.0–12.0 Å^{-1}, dk = 1.0–2.0 Å^{-1}, Hanning or Kaiser–Bessel windowing, and R_{max} = 2.0–10.0 Å, with default values of 3.0–12.0 Å^{-1}, dk = 1.0 Å^{-1}, Hanning windowing, and R_{max} = 6.0 Å. The resulting complex $\chi(R)$ array and its magnitude are subsequently resampled to 928 points using one-dimensional linear interpolation for plotting and export. This final interpolation changes only the number of reported sampling points and does not modify the Fourier transform itself.

Table S1: Parameters defining FEFF EXAFS generation, including their role, typical ranges, and working values used throughout the AI-EXAFS workflow.

Parameter	Role	Typical range	Working value
**Absorber and excitation**			
Absorbing element	Defines the absorbing atom.	Element-specific	Per structure
Absorption edge (K, L_{III}, ...)	Defines the excited core level.	K or L edges	Element-dependent
Amplitude reduction factor (S_0^2)	Accounts for intrinsic many-body losses.	0.7–1.0	Element-specific
**Scattering environment**			
Maximum real-space radius ($R_{\max}$)	Limits the scattering environment.	6–10 Å	10.0 Å
Neighbor inclusion criterion	Defines included scatterers.	All atoms / filtered	All atoms within cutoff
Single-scattering contributions	Includes absorber–neighbor scattering.	Included / excluded	Included
Multiple-scattering contributions	Includes higher-order scattering pathways.	Included / excluded	Included
Maximum EXAFS expansion order	Defines the highest included scattering order.	10–20	20
Maximum total path length	Limits long-path contributions.	6–12 Å	Implicit via $R_{\max}$
**Potential and numerical settings**			
Atomic potential construction	Defines scattering phase shifts and amplitudes.	Default / self-consistent	Self-consistent
Self-consistent field (SCF) radius	Controls the potential convergence region.	3–6 Å	FEFF default
SCF convergence threshold	Controls numerical convergence.	Default / tightened	FEFF default
Energy grid spacing	Defines spectral resolution.	Fixed step size	FEFF default
Momentum-space range ($k_{\min}$, $k_{\max}$)	Defines calculated EXAFS range.	2–15 Å^{-1}	2–12 Å^{-1}

Table S2: FEFF control parameters used for theoretical EXAFS generation together with their functional role and selected working values employed throughout the automated workflow.

Tag	Role	Working value
`CONTROL`	Controls which modules of FEFF are executed.	`1 1 0 0 0 0` – Computes potentials and phase shifts only.
`PRINT`	Controls output verbosity.	`1 0 0 0 0 3` – Outputs absorption cross-section details.
`RMAX`	Sets the maximum radial distance for scattering paths.	`10.0` – Limits the scattering environment to a 10.0 Åradius.
`EXAFS`	Defines the maximum expansion order for EXAFS calculations.	`20.0` – Includes paths up to an EXAFS order of 20.0.

Table S3: Amplitude reduction factors (S_0^2) used for theoretical EXAFS generation, determined from expert-supervised fits to representative experimental metal-foil EXAFS spectra.

Symbol	Phase	Edge	$\mathbf{S_0^2}$
Ti	HCP	K	0.87
V	BCC	K	0.80
Cr	BCC	K	0.69
Mn	BCC	K	0.85
Fe	BCC	K	0.67
Co	HCP	K	0.63
Ni	FCC	K	0.78
Cu	FCC	K	0.90
Zn	HCP	K	0.91
Zr	HCP	K	0.76
Nb	BCC	K	0.82
Mo	BCC	K	0.86
Ru	HCP	K	0.73
Rh	FCC	K	0.78
Pd	FCC	K	0.74
Ag	FCC	K	0.85
In	BCC	K	0.80
Sn	$\beta - Sn$	K	0.96
Sb	P-RHO	K	0.96
Ta	BCC	L3	0.78
W	BCC	L3	0.79
Re	HCP	L3	0.72
Ir	FCC	L3	0.71
Pt	FCC	L3	0.79
Au	FCC	L3	0.85
Pb	FCC	L3	0.74
Bi	RHO	L3	0.67
**Average**			0.80

Table S4: XAS spectral normalization and background subtraction parameters used for standardized EXAFS processing, including definitions, typical ranges, and working values employed throughout the AI-EXAFS workflow.

Parameter	Role	Typical Range	Working Value
**XAS spectral normalization parameters**			
**nvict**	Polynomial order for pre-edge background.	0–3	0
**pre1**	Lower pre-edge fit bound (relative to E_0).	–300 to –10 eV	–200 eV
**pre2**	Upper pre-edge fit bound (relative to E_0).	–50 to 0 eV	–15 eV
**norm1**	Lower post-edge normalization bound.	50–300 eV	150 eV
**norm2**	Upper post-edge normalization bound.	–5 to 5 eV	–1 eV
**make_flat**	Whether to output flattened, normalized $\mu(E)$.	True/False	True
**Background subtraction parameters**			
$r_{\mathbf{bkg}}$	Real-space cutoff for background spline.	0.5–1.5 Å	1.0 Å
E_0	Absorption edge for k conversion.	auto/manual	auto
$k_{\min}$	Minimum k for background fit.	2–4 Å^{-1}	3.0 Å^{-1}
$k_{\max}$	Maximum k for background fit.	10–15 Å^{-1}	12.0 Å^{-1}
$k_{\mathbf{weight}}$	Weight exponent for $\chi(k)$.	1–3	2.0
$k_{\mathbf{step}}$	Increment in k for interpolation.	0.01–0.1 Å^{-1}	0.05 Å^{-1}
**clamp$_{\mathbf{lo}}$**	Low-energy spline constraint weight.	0–2	1.0
**clamp$_{\mathbf{hi}}$**	High-energy spline constraint weight.	0–20	20

Table S5: Fourier-transform parameters used for EXAFS conversion from k-space to R-space, including definitions, typical ranges, and working values employed throughout the AI-EXAFS workflow.

**Parameter**	**Meaning**	**Typical Range**	**Working Value**
$k_{\min}$ **(FT)**	Minimum k for FT window.	2–4 Å^{-1}	3.0 Å^{-1}
$k_{\max}$ **(FT)**	Maximum k for FT window.	10–15 Å^{-1}	12.0 Å^{-1}
$k_{\mathbf{weight}}$ **(FT)**	Weight exponent applied before FT.	1–3	2.0
Δk	Primary taper width for FT window.	2–4	1.50
Δk_2	Secondary taper width (if used).	None / 1–3	None
**window**	Window function used for FT.	Hanning, Kaiser–Bessel	Hanning
$R_{\max,\mathbf{out}}$	Maximum R included in $\chi(R)$ output.	4–8 Å	6.0 Å

Table S6: Hyperparameter optimization space and selected values for the AI-EXAFS graph neural network. Parameters marked with (*) were fixed during optimization.

**Hyperparameter**	**Role**	**Search range**	**Optimized value**
**Node embedding**			
Input node features (*)	Dimension of atomic node features.	92	92
Hidden embedding dimension	Latent node representation size.	92–256	256
Embedding activation	Activation after embedding layers.	ReLU, ELU, LeakyReLU, Softplus, Tanh	LeakyReLU
**Residual block**			
Residual refinement	Use of residual refinement layers.	True / False	True
Number of linear layers	Linear layers in residual block.	0–10	1
Residual activation	Activation within residual layers.	ReLU, ELU, LeakyReLU, Softplus, Tanh	LeakyReLU
**Graph convolution**			
Graph convolution layers	Message-passing depth.	1–10	5
Edge attribute dimension (*)	Dimension of edge features.	1	1
Aggregation scheme (†)	Message aggregation function.	Add, Mean, Max	Max
Batch normalization	Batch normalization after convolutions.	True / False	True
Bias term	Learnable graph convolution bias.	True / False	True
Convolution activation	Activation after convolutions.	ReLU, ELU, LeakyReLU, Softplus, Tanh	LeakyReLU
**Attention pooling**			
Attention heads	Number of pooling heads.	1–20	16
Layer normalization	Normalization after pooling.	True / False	False
Dropout probability	Pooling dropout probability.	0.0–0.8	0.61
**Training**			
Batch size	Graphs per optimization step.	6–128	32
Learning rate	Optimization step size.	1×10^{-6}–1×10^{-2}	0.0005860
Weight decay	L2 penalty on network weights.	1×10^{-8}–1×10^{-3}	1×10^{-8}

(†) Aggregation scheme refers to the message-passing operation combining neighbor messages at each node within a graph convolution layer. It is distinct from the global pooling operation that aggregates node embeddings into a single graph-level representation, which uses multi-head attention (**Table S7**).

Table S7: Ablation analysis of the AI-EXAFS framework showing average EXAFS R-factor and mean absolute error (MAE) values computed in k-space for representation, architectural, and training-objective ablations. Arrows in parentheses indicate the variation relative to the reference model.

**Ablation**	**Avg. R-factor**	**Avg. MAE**	**Δ R-factor**[(a)]	**Δ MAE**[(a)]
**Graph representation**				
Removed edge distances	0.392	0.161	8.123 (↑)	1.872 (↑)
Removed Absorber-centered connections (AC)	0.074	0.070	0.712 (↑)	0.254 (↑)
**Model architecture**				
Removed residual block	0.053	0.060	0.243 (↑)	0.068 (↑)
Add pooling instead of attention[(b)]	0.048	0.056	0.124 (↑)	-0.001 (∼)
Mean pooling instead of attention	0.050	0.057	0.163 (↑)	0.023 (↑)
Max pooling instead of attention	0.102	0.080	1.377 (↑)	0.430 (↑)
Removed sigmoid output activation[(b)]	0.061	0.064	0.412 (↑)	0.139 (↑)
**Training objective**				
MAE loss instead of R-factor	0.049	0.056	0.129 (↑)	0.009 (∼)
MSE loss instead of R-factor	0.058	0.062	0.338 (↑)	0.106 (↑)
SID loss instead of R-factor	0.084	0.080	0.964 (↑)	0.426 (↑)
**Reference model**				
Full AI-EXAFS model	0.043 (0.019)	0.056 (0.027)	–	–

(a) Relative changes computed as $\Delta = (x_{\text{ablation}} - x_{\text{reference}})/x_{\text{reference}}$.
(b) Training instability associated with NaN values during optimization.

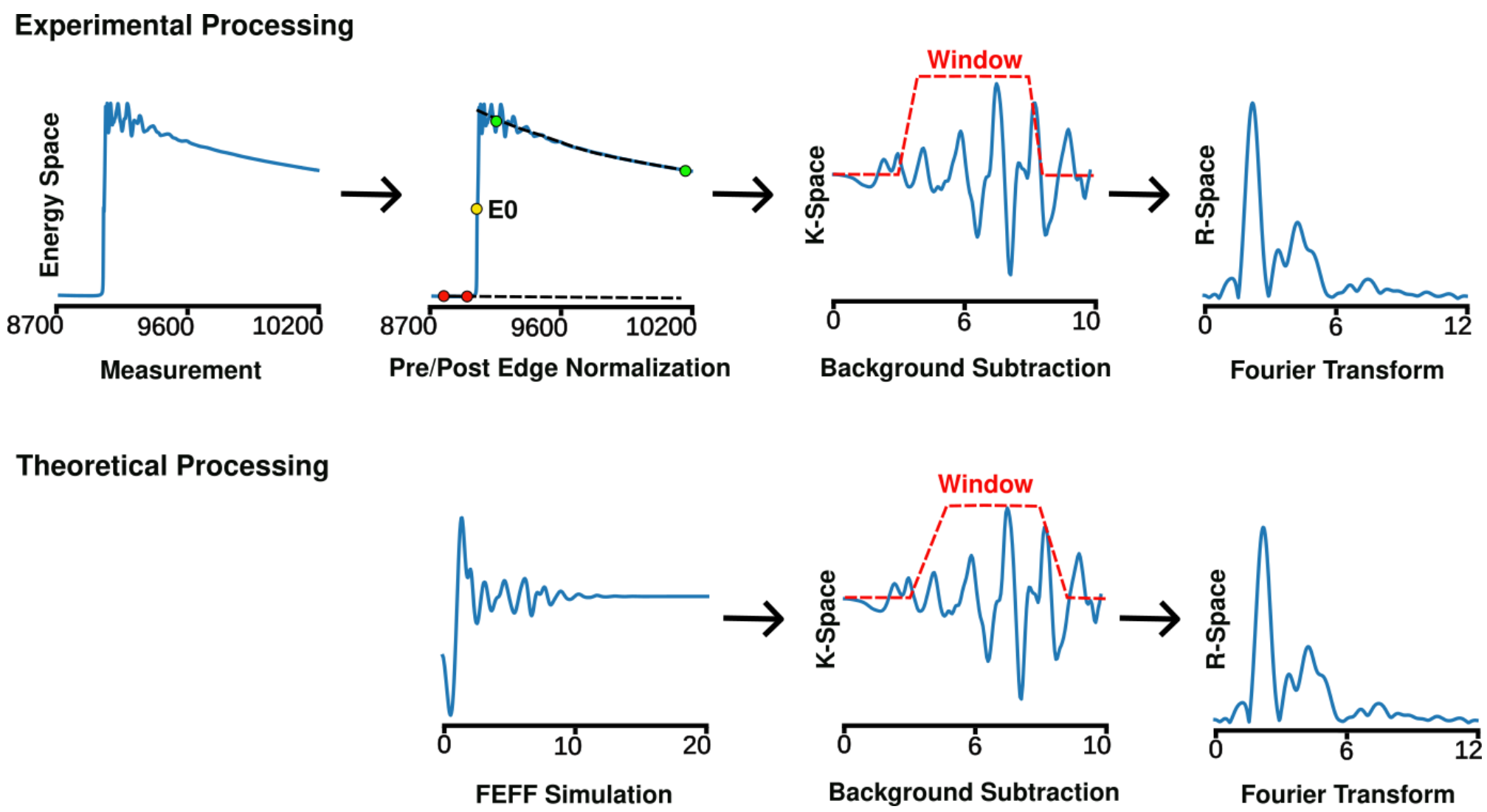

Figure S1: EXAFS spectral processing workflow. Experimental and theoretical EXAFS spectra undergo a sequence of processing steps including energy calibration, XAS spectral normalization, background subtraction, windowing, and Fourier transformation prior to analysis and comparison. Variations in these procedures and parameter choices can introduce differences across workflows and users, motivating the standardized processing protocol adopted throughout the present work.

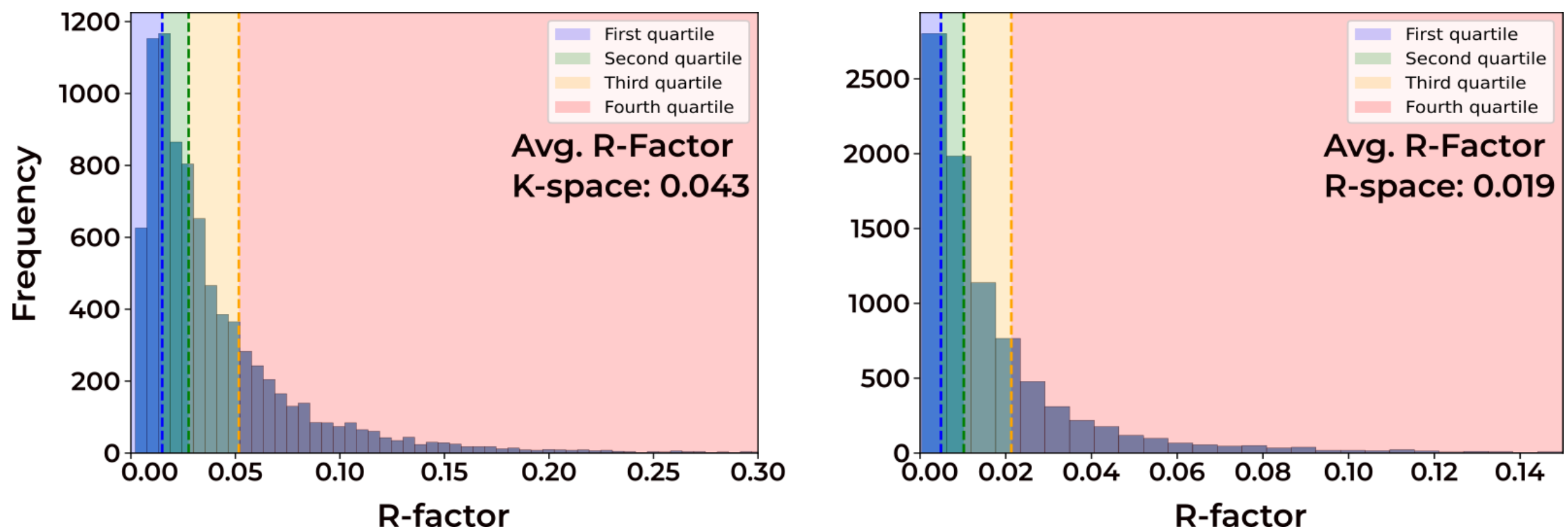

Figure S2: Distribution of model prediction errors across the test set. Histograms of EXAFS R-factor values computed for the test-set predictions in k-space and R-space. The distributions illustrate the overall predictive performance and variability of the AI-EXAFS model across chemically diverse coordination environments.

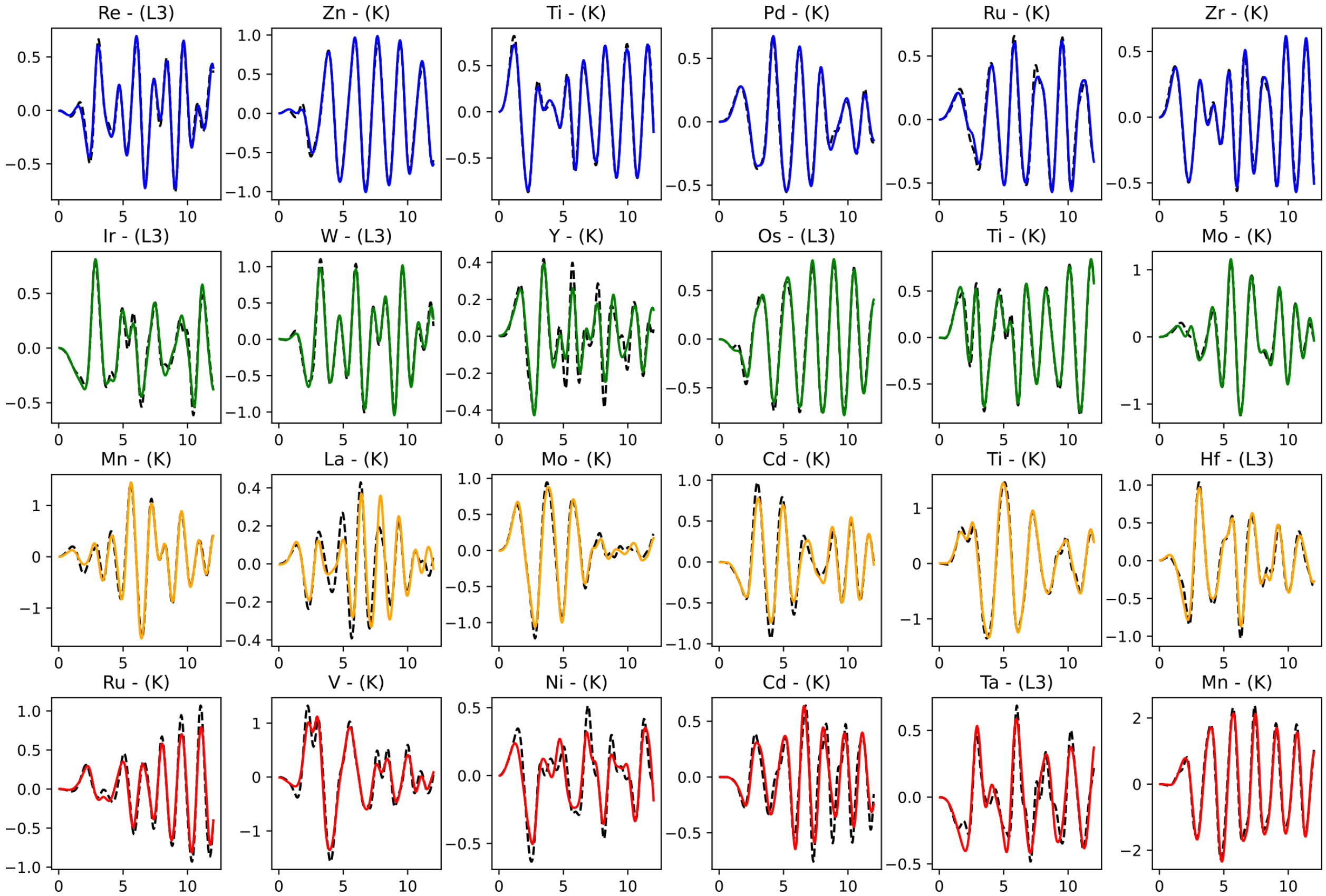

Figure S3: Representative EXAFS predictions in k-space. Comparison between reference and AI-EXAFS-predicted $\chi(k)$ ($\chi(k) \cdot k^2$) spectra for representative test-set systems spanning different absorbing elements and coordination environments. The model accurately reproduces oscillation phase, relative amplitude modulation, and interference features across the k-range.

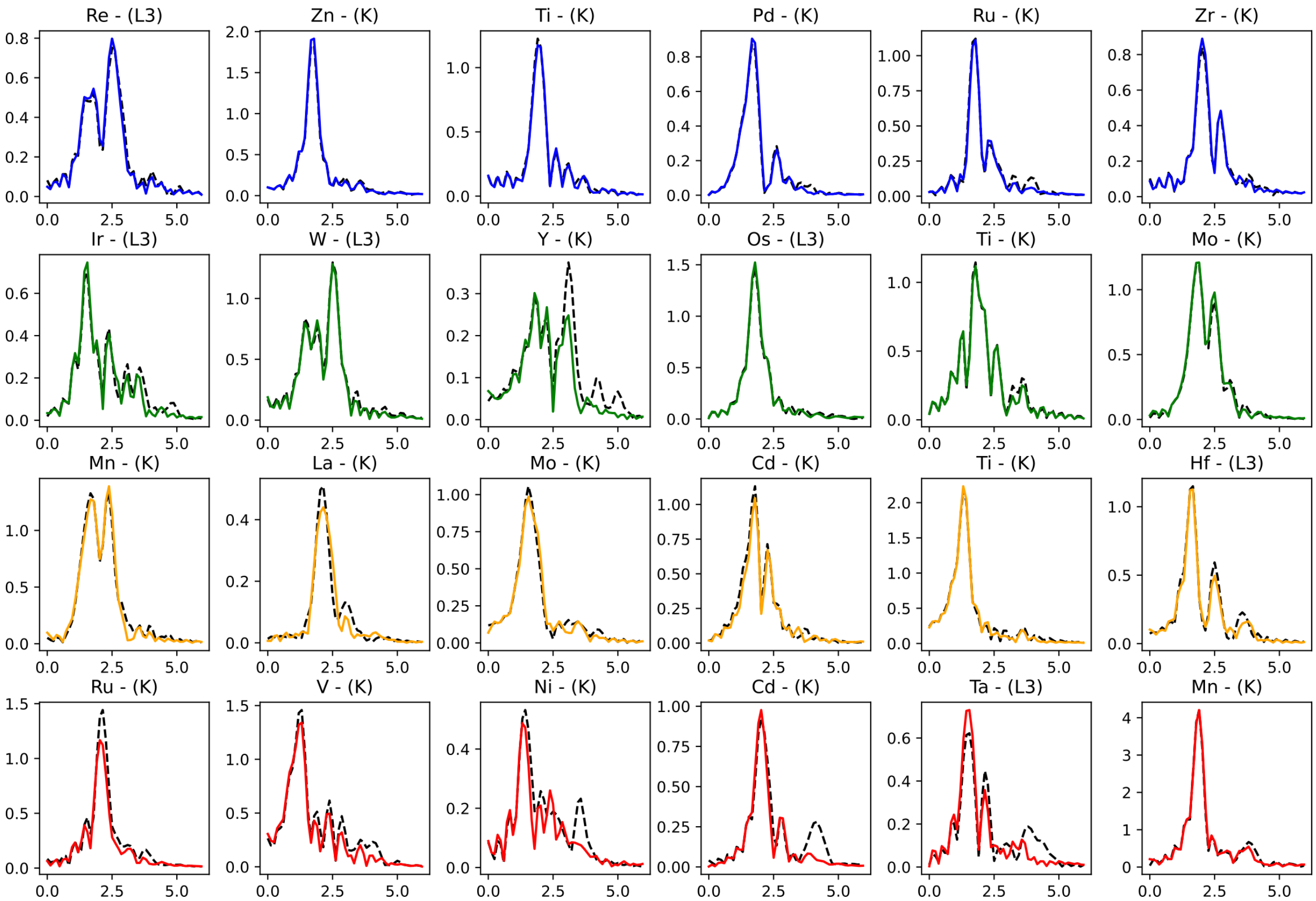

Figure S4: Representative EXAFS predictions in R-space. Fourier-transformed EXAFS spectra corresponding to the representative k-space predictions shown in **Figure S3**. AI-EXAFS accurately reproduces coordination-shell peak positions, relative peak intensities, and medium-range structural features in R-space.

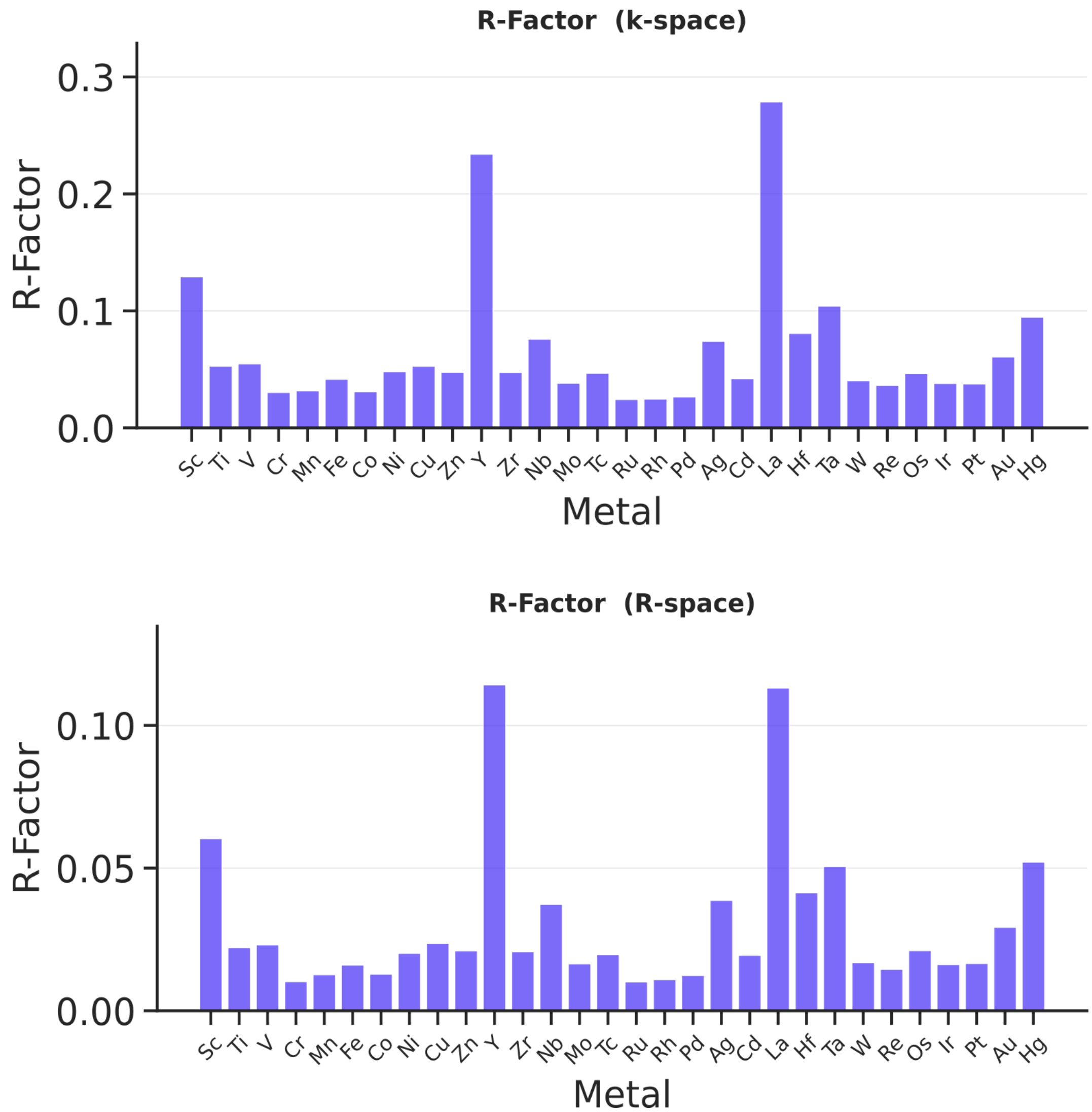

Figure S5: Element-resolved prediction error across the test set. Average EXAFS R-factor values computed in both k- and R-space for each absorbing element. Bars report the mean prediction error over all test-set spectra associated with each element, providing an element-resolved assessment of model performance across distinct scattering regimes. Lower R-factor values indicate closer agreement between predicted and reference EXAFS spectra.

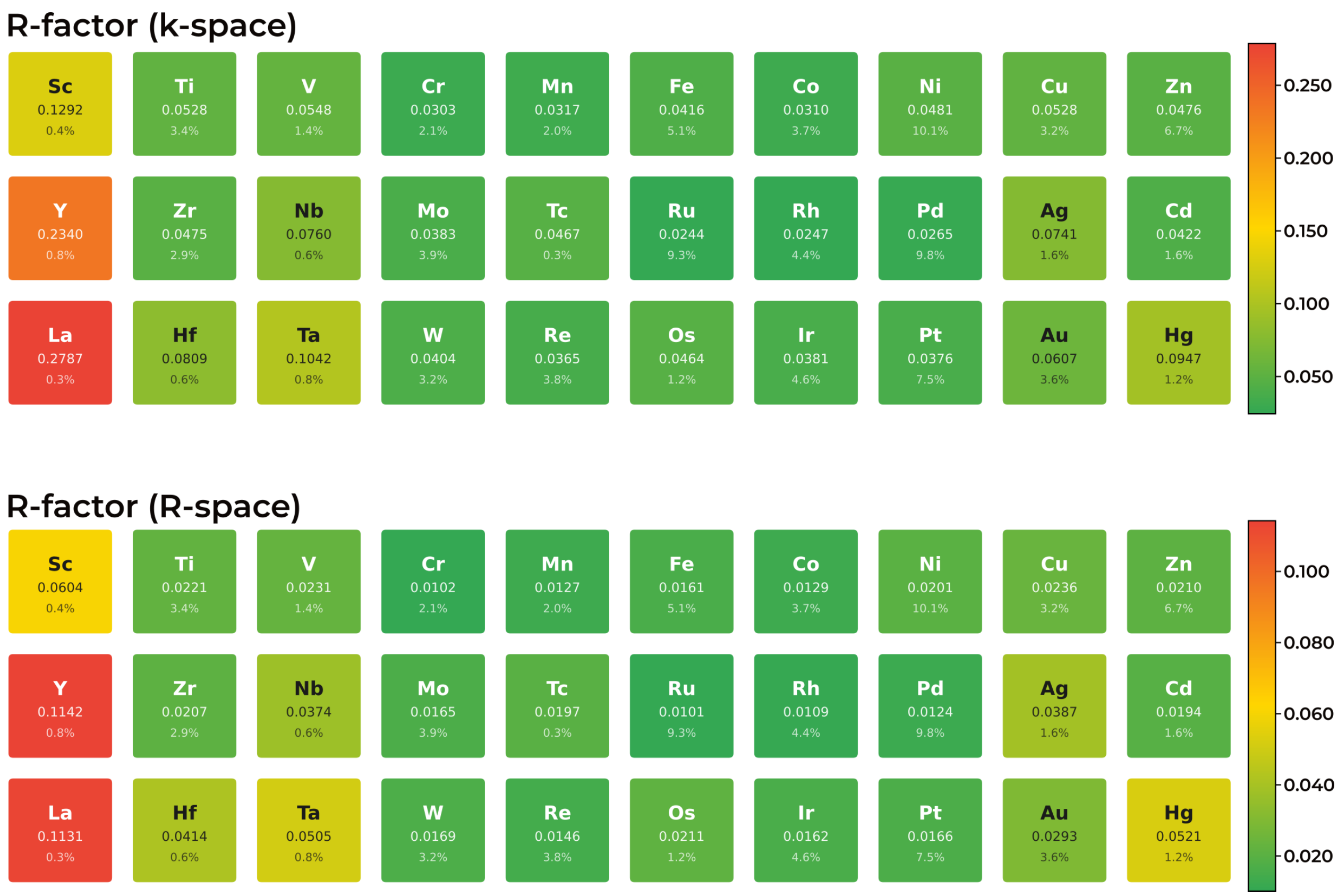

Figure S6: EXAFS reconstruction performance per absorbing atom. Average R-factor values computed in both k-space and R-space for each absorbing atom in the held-out test set. Each tile reports the element symbol, the corresponding mean R-factor, and the percentage of test-set structures containing that absorber. The color scale encodes the R-factor, with greener tiles indicating lower reconstruction error and yellow-to-red tiles indicating larger deviations.

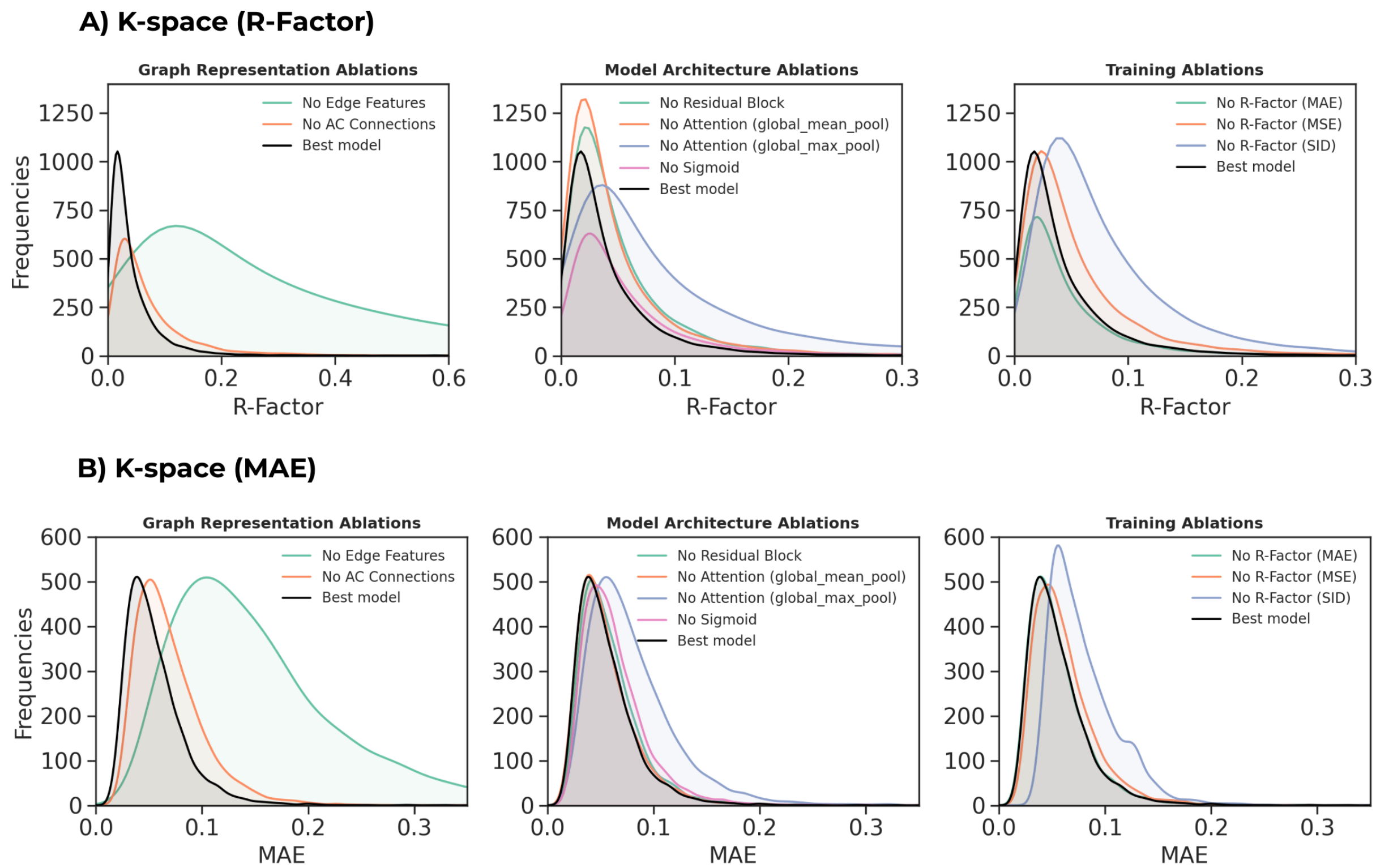

Figure S7: Distribution of prediction errors across ablation variants. Frequency distributions of EXAFS R-factor and MAE values computed in k-space for graph representation, architectural, and training-objective ablations. AC denotes absorber-centered connections. The full AI-EXAFS model (black) is shown as reference in each panel.

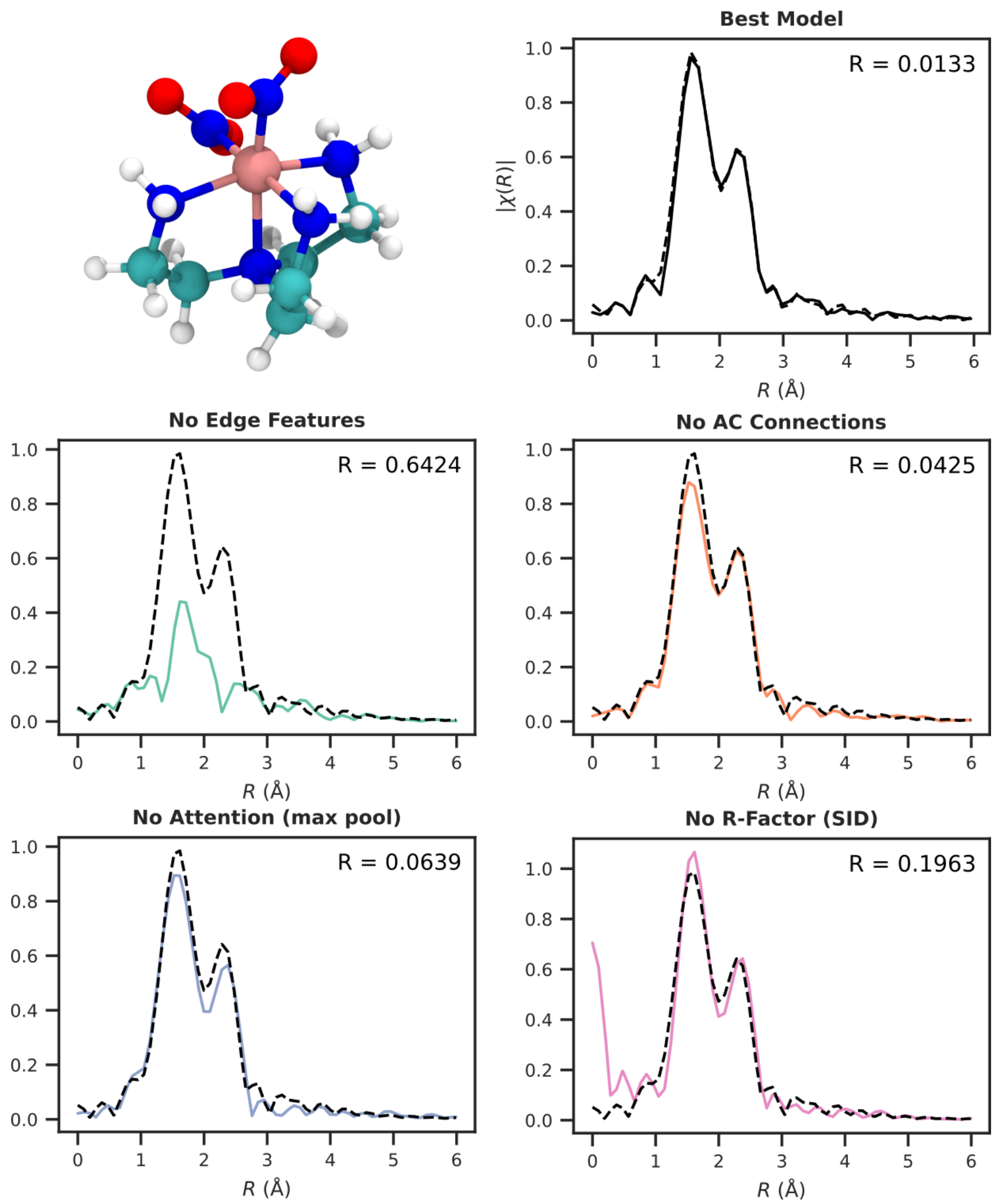

Figure S8: Representative ablation analysis of a Co K-edge EXAFS prediction in R-space. Comparison between reference spectra (black dashed curves) and predictions obtained using the full AI-EXAFS model and representative ablated variants (solid colored curves). Panels show the Fourier-transformed magnitude, $|\chi(R)|$. AC denotes absorber-centered connections. Reported R-factor values correspond to each prediction.

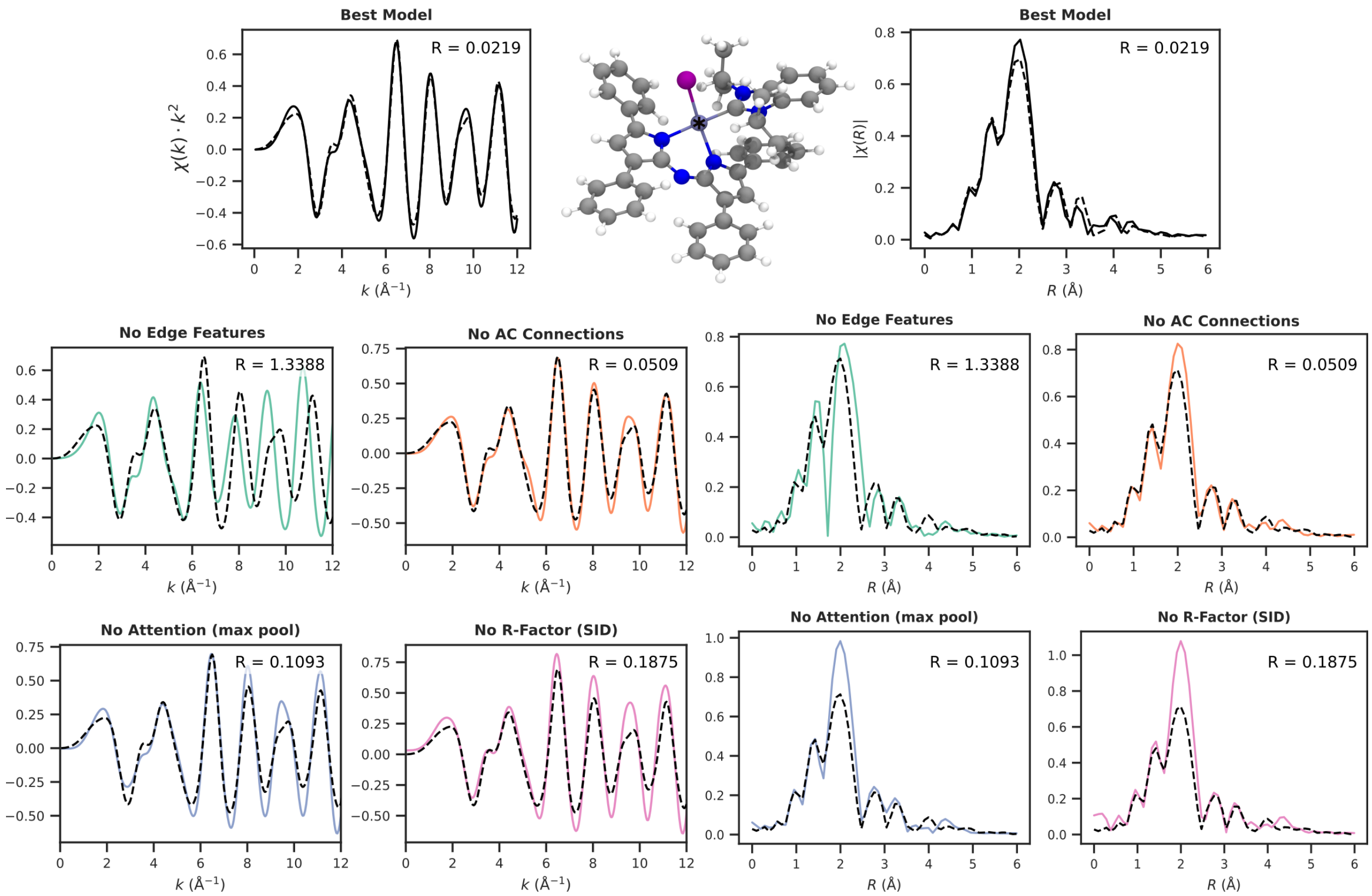

Figure S9: Representative ablation analysis of a Pd K-edge EXAFS prediction in k-space and R-space. Comparison between reference spectra (black dashed curves) and predictions obtained using the full AI-EXAFS model and representative ablated variants (solid colored curves). Left panels show $\chi(k)$ ($\chi(k) \cdot k^2$) spectra in k-space, while right panels show the corresponding Fourier-transformed magnitude, $|\chi(R)|$. AC denotes absorber-centered connections. Reported R-factor values correspond to each prediction.

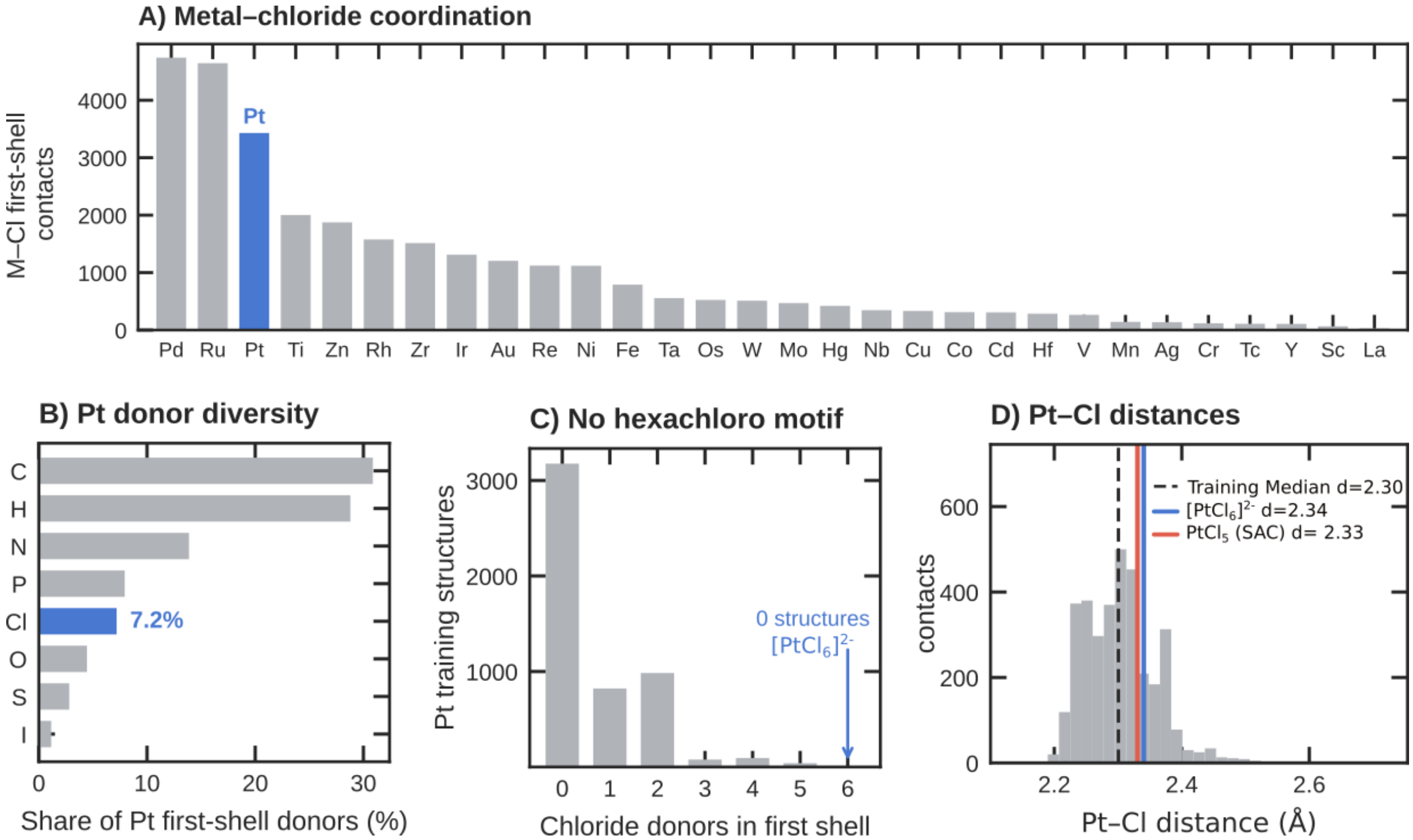

Figure S10: Representation of Pt–Cl environments in the AI-EXAFS training set. (A) Number of first-shell chloride contacts for each absorbing element across the training set, with Pt highlighted. (B) Distribution of first-shell donor elements within the Pt training subset. (C) Number of Pt training structures as a function of the number of first-shell chloride donors, showing that no training structure contains the complete $[PtCl_6]^{2-}$ coordination motif. (D) Distribution of first-shell Pt–Cl distances, with the training median (dashed line) and the mean Pt–Cl distances of $[PtCl_6]^{2-}$ and the supported $PtCl_5$ catalyst (solid lines) indicated. The first coordination shell is defined using a 3.0 Å cutoff; further details are provided in **Note S8**.

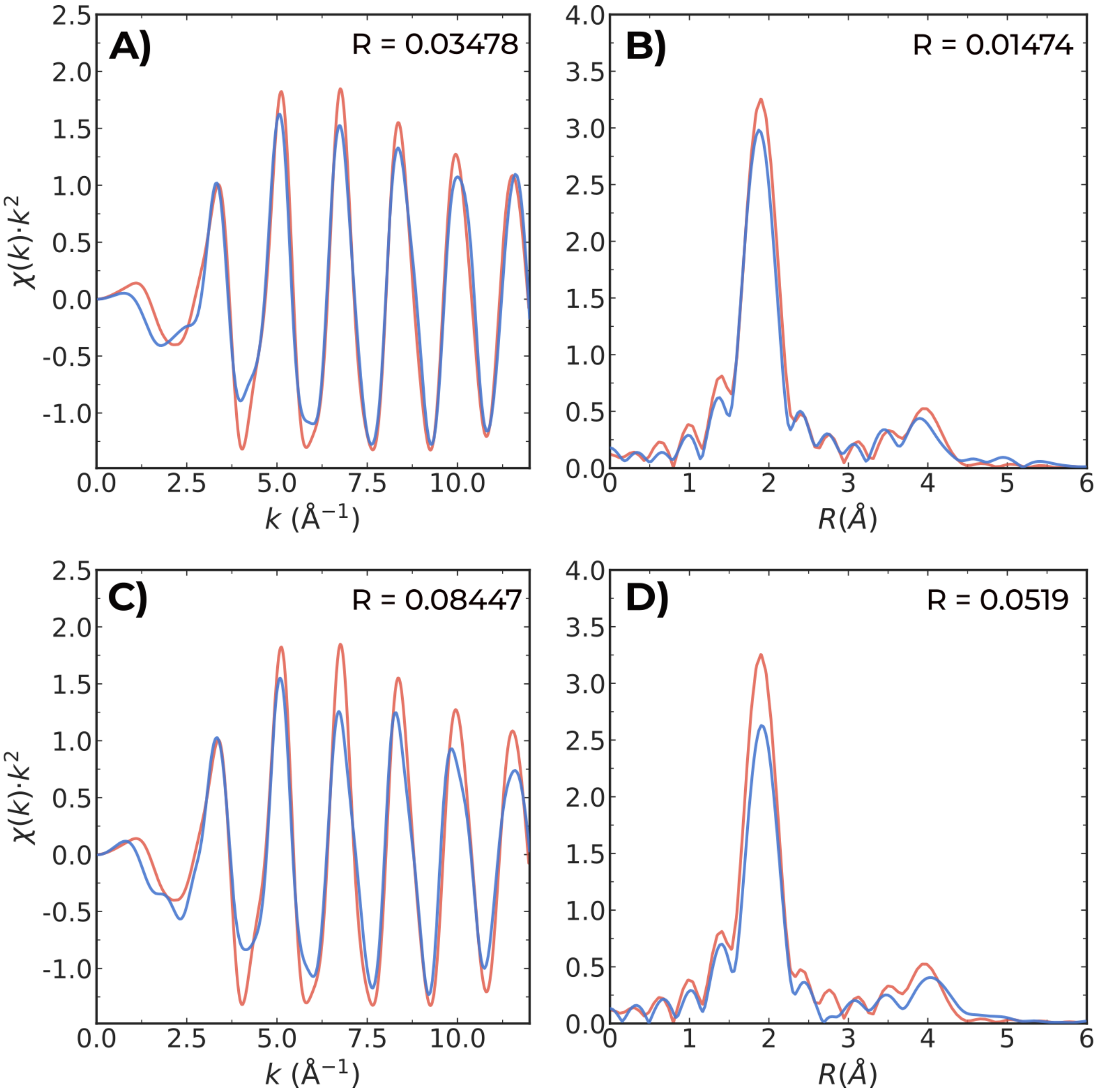

Figure S11: Stress test of AI-EXAFS after removing highly similar training environments for $[PtCl_6]^{2-}$. Comparison between the FEFF reference spectrum (red) and AI-EXAFS prediction (blue) in k-space (left) and R-space (right). (A–B) Prediction from the model trained on the 68,791-structure training subset. (C–D) Prediction after removal of training environments with SOAP similarity greater than or equal to 0.80 to either experimental Pt system and model retraining. Removal of highly similar environments increases the R-factor while retaining the principal spectral features in both k- and R-space. Details of the SOAP analysis and retraining protocol are provided in **Note S8**.

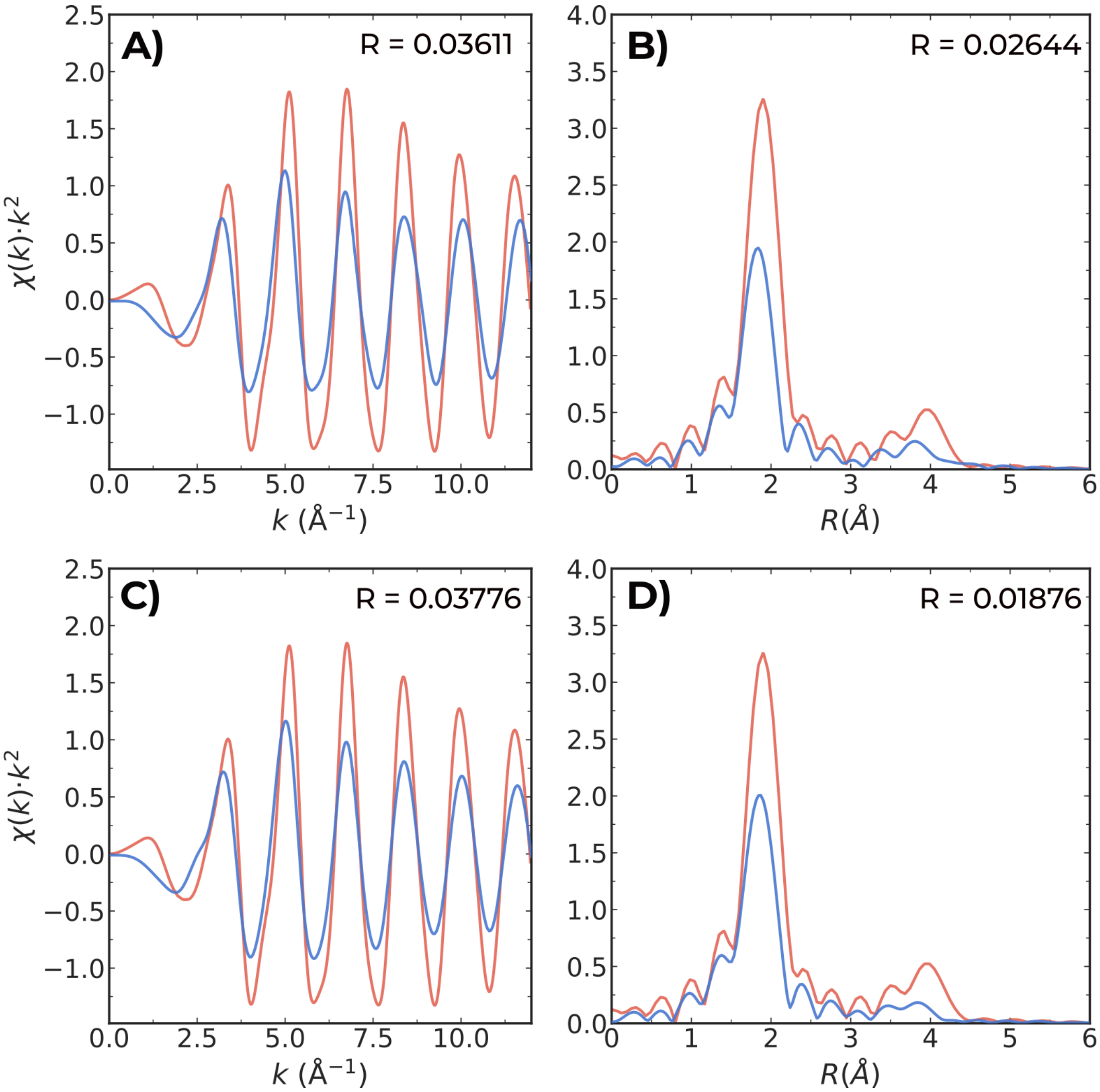

Figure S12: Stress test of AI-EXAFS after removing highly similar training environments for the supported $PtCl_5$ single-atom catalyst. Comparison between the FEFF reference spectrum (red) and AI-EXAFS prediction (blue) in k-space (left) and R-space (right). (A–B) Prediction from the model trained on the 68,791-structure training subset. (C–D) Prediction after removal of training environments with SOAP similarity greater than or equal to 0.80 to either Pt test environment and model retraining. The prediction remains largely unchanged after removal of the highly similar environments, with the principal spectral features retained in both k- and R-space. Details of the SOAP analysis and retraining protocol are provided in **Note S8**.

References

(S1) Ong, S. P.; Richards, W. D.; Jain, A.; Hautier, G.; Kocher, M.; Cholia, S.; Gunter, D.; Chevrier, V. L.; Persson, K. A.; Ceder, G. Python Materials Genomics (pymatgen): A robust, open-source python library for materials analysis. *Computational Materials Science* **2013**, *68*, 314–319.

(S2) Hjorth Larsen, A.; Jørgen Mortensen, J.; Blomqvist, J.; Castelli, I. E.; Christensen, R.; Dułak, M.; Friis, J.; Groves, M. N.; Hammer, B.; Hargus, C.; Hermes, E. D.; Jennings, P. C.; Bjerre Jensen, P.; Kermode, J.; Kitchin, J. R.; Leonhard Kolsbjerg, E.; Kubal, J.; Kaasbjerg, K.; Lysgaard, S.; Bergmann Maronsson, J.; Maxson, T.; Olsen, T.; Pastewka, L.; Peterson, A.; Rostgaard, C.; Schiøtz, J.; Schütt, O.; Strange, M.; Thygesen, K. S.; Vegge, T.; Vilhelmsen, L.; Walter, M.; Zeng, Z.; Jacobsen, K. W. The atomic simulation environment—a Python library for working with atoms. *Journal of Physics: Condensed Matter* **2017**, *29*, 273002.

(S3) Isayev, O.; Oses, C.; Toher, C.; Gossett, E.; Curtarolo, S.; Tropsha, A. Universal fragment descriptors for predicting properties of inorganic crystals. *Nature communications* **2017**, *8*, 15679.

(S4) Fey, M.; Lenssen, J. E. Fast graph representation learning with PyTorch Geometric. *arXiv preprint arXiv:1903.02428* **2019**,

(S5) Newville, M. Larch: An analysis package for XAFS and related spectroscopies. Journal of Physics: Conference Series. 2013; p 012007.

(S6) Ravel, B.; Newville, M. ATHENA, ARTEMIS, HEPHAESTUS: data analysis for X-ray absorption spectroscopy using IFEFFIT. *Journal of synchrotron radiation* **2005**, *12*, 537–541.

(S7) Ressler, T. WinXAS: a program for X-ray absorption spectroscopy data analysis under MS-Windows. *Synchrotron Radiation* **1998**, *5*, 118–122.

(S8) Kas, J. J.; Vila, F.; Rehr, J. The FEFF code. *International Tables for Crystallography* **2020**, *1*.

(S9) Jain, A.; Ong, S. P.; Chen, W.; Medasani, B.; Qu, X.; Kocher, M.; Brafman, M.; Petretto, G.; Rignanese, G.-M.; Hautier, G.; Gunter, D.; Persson, K. A. FireWorks: a dynamic workflow system designed for high-throughput applications. *Concurrency and Computation: Practice and Experience* **2015**, *27*, 5037–5059.

(S10) Mathew, K.; Montoya, J. H.; Faghaninia, A.; Dwarakanath, S.; Aykol, M.; Tang, H.; heng Chu, I.; Smidt, T.; Bocklund, B.; Horton, M.; Dagdelen, J.; Wood, B.; Liu, Z.-K.; Neaton, J.; Ong, S. P.;

Persson, K.; Jain, A. Atomate: A high-level interface to generate, execute, and analyze computational materials science workflows. *Computational Materials Science* **2017**, *139*, 140–152.

(S11) Henry, G. Howard chu on lightning memory-mapped database. *Ieee Software* **2019**, *36*, 83–87.

(S12) Lee, J.; Lee, Y.; Kim, J.; Kosiorek, A.; Choi, S.; Teh, Y. W. Set transformer: A framework for attention-based permutation-invariant neural networks. International conference on machine learning. 2019; pp 3744–3753.

(S13) Vaswani, A.; Shazeer, N.; Parmar, N.; Uszkoreit, J.; Jones, L.; Gomez, A. N.; Kaiser, Ł.; Polosukhin, I. Attention is all you need. *Advances in neural information processing systems* **2017**, *30*.

(S14) Kingma, D. P.; Ba, J. Adam: A method for stochastic optimization. *arXiv preprint arXiv:1412.6980* **2014**,

(S15) Calvin, S. *XAFS for Everyone*; CRC press, 2024.

(S16) Watanabe, S. Tree-structured parzen estimator: Understanding its algorithm components and their roles for better empirical performance. *arXiv preprint arXiv:2304.11127* **2023**,

(S17) Akiba, T.; Sano, S.; Yanase, T.; Ohta, T.; Koyama, M. Optuna: A Next-generation Hyperparameter Optimization Framework. Proceedings of the 25th ACM SIGKDD International Conference on Knowledge Discovery and Data Mining. 2019.

(S18) Sayers, D. Report of the International XAFS Society Standards and Criteria Committee. *the IXS Standards and Criteria Committee* **2000**,

(S19) Paszke, A.; Gross, S.; Massa, F.; Lerer, A.; Bradbury, J.; Chanan, G.; Killeen, T.; Lin, Z.; Gimelshein, N.; Antiga, L.; Desmaison, A.; Kopf, A.; Yang, E.; DeVito, Z.; Raison, M.; Tejani, A.; Chilamkurthy, S.; Steiner, B.; Fang, L.; Bai, J.; Chintala, S. Pytorch: An imperative style, high-performance deep learning library. *Advances in neural information processing systems* **2019**, *32*.

(S20) Falcon, W. Pytorch lightning. 2019; https://doi.org/10.5281/zenodo.3828935.

(S21) Yadan, O. Hydra - A framework for elegantly configuring complex applications. Github, 2019; https://github.com/facebookresearch/hydra.

(S22) Biewald, L. Experiment Tracking with Weights and Biases. 2020; https://www.wandb.com/, Software available from wandb.com.

(S23) Himanen, L.; Jäger, M. O.; Morooka, E. V.; Canova, F. F.; Ranawat, Y. S.; Gao, D. Z.; Rinke, P.; Foster, A. S. DScribe: Library of descriptors for machine learning in materials science. *Computer Physics Communications* **2020**, *247*, 106949.

(S24) Khorasani, M.; Abdou, M.; Fernández, J. H. *Streamlit for Web Development: Build and Scale Secure Python-Powered Apps with Streamlit*; Springer, 2025.

(S25) Rego, N.; Koes, D. 3Dmol.js: molecular visualization with WebGL. *Bioinformatics* **2015**, *31*, 1322–1324.
